# Development of Mechanistic Neural Mass (mNM) Models that Link Physiology to Mean-Field Dynamics

**DOI:** 10.3389/fnetp.2022.911090

**Published:** 2022-09-28

**Authors:** Richa Tripathi, Bruce J. Gluckman

**Affiliations:** ^1^ Center for Neural Engineering, The Pennsylvania State University, University Park, PA, United States; ^2^ Indian Institute of Technology Gandhinagar, Gandhinagar, India; ^3^ Center for Advanced Systems Understanding (CASUS), HZDR, Görlitz, Germany; ^4^ Departments of Engineering Science and Mechanics, Biomedical Engineering, The Pennsylvania State University, University Park, PA, United States; ^5^ Department of Neurosurgery, College of Medicine, The Pennsylvania State University, Hershey, PA, United States

**Keywords:** excitation-inhibition imbalance, depolarization block, neural mass models, brain networks and dynamic connectivity, pathophysiology

## Abstract

Brain rhythms emerge from the mean-field activity of networks of neurons. There have been many efforts to build mathematical and computational embodiments in the form of discrete cell-group activities—termed neural masses—to understand in particular the origins of evoked potentials, intrinsic patterns of activities such as theta, regulation of sleep, Parkinson’s disease related dynamics, and mimic seizure dynamics. As originally utilized, standard neural masses convert input through a sigmoidal function to a firing rate, and firing rate through a synaptic alpha function to other masses. Here we define a process to build mechanistic neural masses (mNMs) as mean-field models of microscopic membrane-type (Hodgkin Huxley type) models of different neuron types that duplicate the stability, firing rate, and associated bifurcations as function of relevant slow variables - such as extracellular potassium - and synaptic current; and whose output is both firing rate and impact on the slow variables - such as transmembrane potassium flux. Small networks composed of just excitatory and inhibitory mNMs demonstrate expected dynamical states including firing, runaway excitation and depolarization block, and these transitions change in biologically observed ways with changes in extracellular potassium and excitatory-inhibitory balance.

## 1 Introduction

Neural masses are mean field models of neural cell group activities. Neural mass models were first introduced by Walter Freeman in the 1970s (Freeman, 1972a; Freeman, 1975) as a model to relate the activity, represented by the field potentials or EEG from one brain region to the EEG in another region, without the intricate details of neuron-level connectivity and action-potential dynamics and timing.

In the past, networks of NMs (neural mass models NMMs) have been constructed and used to understand the origins of evoked potentials (Jansen et al., 1993; Jansen and Rit, 1995), intrinsic patterns of activities such as theta (Segneri et al., 2020), regulation of sleep (Diniz Behn and Booth, 2010; Fleshner et al., 2011; Bhattacharya, 2013; Costa et al., 2016; Schellenberger Costa et al., 2016), Parkinson’s disease related dynamics (Liu et al., 2016; Liu et al., 2017; Basu et al., 2018; Liu et al., 2021), and instabilities such as seizure dynamics (Wendling et al., 2002; Wendling, 2008; Aarabi and He, 2014; López-Cuevas et al., 2015; Kameneva et al., 2017; Köksal Ersöz et al., 2020). But their firing dynamics are disconnected from real physiological parameters and instead are assumed to be sigmoidal functions of their summed input.

It is our objective here to build discrete mean-field model elements - neural masses (NM) - whose dynamics, detailed parameters, and interactions with each other and their environment can be linked quantitatively across scales directly to membrane-level mechanics of single neurons. Such mechanistic neural masses can then be used to build network models that interact with the environment and have measures that can quantitatively be related to physiological measures. Our interest here is especially to embody some of the underlying physiological mechanics that are hypothesized to be involved in epileptic dynamics.

Epilepsy - the occurrence of recurrent spontaneous seizures - is a major human health concern, leading to significant deficit in quality of life, higher mortality, and significant economic impact. In developed countries, epilepsy rates are close to 1% of the population. There are pharmacological interventions, but these provide successful abatement of seizures in only as few as 2/3 of patients, and often have significant side effects (Löscher and Schmidt, 2011; Svalheim et al., 2015; Meador and Loring, 2016; Chen et al., 2017).

At the network level, seizure susceptibility is often described as resulting from an excitatory-inhibitory imbalance. At the cellular level, this can mechanistically result from transient inactivation of inhibitory neurons leading to runaway excitation (McCormick and Contreras, 2001).

Pre- or post-seizure side effects or related neurological phenomena include postictal generalized EEG suppression (PGES) (Somjen, 2004; Poh et al., 2012; Rajakulendran and Nashef, 2015), post-ictal amnesia (Corcoran and Thompson, 1992; Butler et al., 2007; Lanzone et al., 2018), and sudden unexplained death in epilepsy (SUDEP) (Ryvlin et al., 2013; Rajakulendran and Nashef, 2015; Noebels, 2019). Mortality in persons with epilepsy are nearly two times that of the population at large (Thurman et al., 2017). In 2015, Aiba and Nobels demonstrated that spreading depolarization (SD) invading the brainstem could cause autonomic shutdown, and therefore might be one of the mechanisms of SUDEP (Aiba and Noebels, 2015), and once looked for, spontaneous seizure-associated SD has been observed in animal models of epilepsy (Ssentongo et al., 2017; Bahari et al., 2018; Loonen et al., 2019).

Leão discovered spreading depolarization - which he denoted spreading depression - in 1944 in the context of acute induced seizure models (Leao, 1944; Leo, 1944; Leo and Morison, 1945). SD is readily-associated with a range of other neurological disorders including migraine, stroke, sub-arachnoid hemorrhage, and traumatic brain injury (Pietrobon and Moskowitz, 2014; Brennan and Pietrobon, 2018; Tolner et al., 2019).

Although many mechanisms can contribute to SD, the fundamental component is that elevation of the extracelluar potassium concentration leads individual neurons into depolarization block (DB) - a state in which the potassium channels are substantially open, but the potassium current is not enough to repolarize the membrane potential and restore the channels to the normally closed state. In this state, the potassium flux can further increase the extracellular potassium concentration and, through diffusion, induce nearby neurons into DB (Somjen, 2004).

These mechanics are well known and captured in membrane and compartment models of single neurons (Kager et al., 2000; Wei et al., 2014).

In this work we define a process to develop mechanistic neural mass (mNM) elements derived from single-cell membrane dynamics. Functionally, these mass elements should serve as plug-in replacements for commonly-used elements in existing neural mass models. These differ in that they parametrize state transitions of the single-cell models to include depolarization block, their sensitivity to slow variables such as extracellular potassium and accommodation state, and their coupling back to those slow variables. We find that small excitatotry-inhibitory networks built from those networks embody dynamical transitions that underlie seizure and spreading depolarization dynamics.

It is our expectation that network models constructed with the resulting mechanistic neural masses (mNMs) we develop can reproduce normal dynamics as well as the pathological dynamics of seizures and SD in a way that can be linked to measurable biological features such as extracellular potassium, make quantitative predictions about their effects, and meaningfully give insight as to why they have such effects. As an example, we aim to give insight as to why a modest change in excitatory-inhibitory balance yields a network that is not continually seizing or undergoing SD but is significantly more susceptible to these instabilities.

As a primary step, we outline the procedure for deriving mechanistic neural masses and demonstrate it for two canonical neuron types: a non-accommodating mammalian inhibitory neuron, based on the Wang-Buzsáki (WB) neuron (Wang and Buzsáki, 1996), and an excitatory one with accommodation based on the Pinsky-Rinzel (PR) pyramidal neuron (Pinsky and Rinzel, 1994).

**FIGURE 1:**
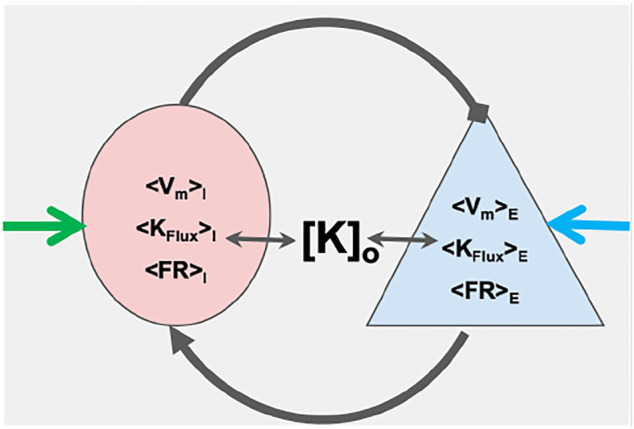
Schematic of coupled masses: The figure shows a couplet of an excitatory (blue) and an inhibitory (pink) mass. The two masses represent mean dynamics of ensemble of respective neuron types in terms of mean firing rates (⟨FR⟩), mean potassium ion flux (⟨K_*Flux*_⟩) and mean membrane potential (⟨V_*m*_⟩). The means of these dynamical quantities for the excitatory(E) and inhibitory (I) masses are estimated from the canonical excitatory and the inhibitory neuron models, respectively [*K*]_*o*_ is the potassium concentration of the common extracellular bath of the masses. The masses couple to each other through their firing with specific coupling strengths and interact with extracellular potassium ion bath. External inputs are represented by blue and green arrows.

We then demonstrate that a two-mass inhibitory-excitatory network built from these elements, illustrated in Figure 1 already express the range of normal balanced firing, runaway excitation and depolarization block that form the fundamentals of normal and pathological dynamics for transitions into seizure and SD, and how changes in extracellular potassium modifies these transitions. Furthermore, small changes in static excitatory-inhibitory balance, as might occur from interneuron damage, significantly change the susceptibility of this network to such transitions - explicilty making the network more sensitive to small changes in extracellular potassium. We leave for future work larger networks and coupling these mNMs to the extracellular space and its ion concentrations and to glial networks.

## 2 Methods

Neural masses (Freeman, 1972a; Freeman, 1972b; Wilson and Cowan, 1972; Lopes da Silva et al., 1974; Freeman, 1975; Jansen and Rit, 1995; Cona et al., 2014) are simplifications of mean-field dynamics that are thought to represent the input-output dynamics of a sub-population of neurons. In NMMs, the input to one NM is a sum over the output from the other masses. Classically, the output of a NM is its firing rate (FR), and its contribution to input on downstream masses is the FR convolved in time with a synaptic response function, described by an alpha function (Rall et al., 1967), to yield an instantaneous input current. Classically, the FR is taken as a sigmoidal function of the total input current.

Neural mass dynamics are supposed to represent the activity of groups of hundreds to millions of neurons (Freeman, 1972a). While this could be done by integrating a like number of membrane-type models, it is quite computationally intensive. Alternately, one could replace the mass with a small number - or even one - membrane-type model, but the resulting output would suffer from the precise action potential timing of the elements which would dominate the dynamics.

Critical in the process of creating neural masses is that the dynamics are significantly simplified from the detailed firing/action potential dynamics of Hodgkin-Huxley type membrane dynamics. The simplifications achieved with neural mass dynamics are very low computational load, and loss of action potential timing sensitivity by conversion to firing rates.

To achieve this mean field approach from Hodgkin-Huxley type single neuron models - to create mechanistic neural masses - we utilize a time-scale separation approach in which the fast dynamics associated with the action potentials are separated from slower dynamics by treating those other dynamics as quasi-static. We then parameterize the dynamics of that fast model, explicitly fitting as output the firing rate, mean membrane potential, and potassium flux, as a function of those quasi-static inputs. These outputs are then used in the evolution dynamics of the slow input variables, as well as the coupling of the mass to its environment and to other masses.

### 2.1 Procedure to Obtain Mechanistic Neural Mass

A key idea behind the development of mNMs, is that the time-averages of the fast dynamics of the neuron models can be expressed as simple functions of the total current and quasi-static (slow) state variables. Hence, instead of looking at detailed firing response of neurons in terms of instantaneous changes in membrane potentials, potassium fluxes and firing rates, we look steady-state response of the neuron models and use them to characterize the corresponding neural masses. These steady state responses are then parametrized (fitted) with simple analytic functions of the net input current and the slow state variables. These analytical functions fully characterize our neural mass outputs in terms of mean firing rate, mean membrane potential, and mean potassium flux.

We are interested in dynamics of the masses in the bifurcation space of injected current and changes in the transmembrane potassium Nernst potential (*ν*_*K*_) from its nominal value. If the nominal and actual Nernst potentials are ${\hat{\nu }}_{K}$ and *ν*_*K*_, respectively, the change in it is expressed as Δ*ν*_*K*_.

$$\begin{array}{l} \Delta \nu_{K}=\nu_{K}-\hat{\nu_{K}}\\ =\frac{RT}{F}\left[\ln\left(\frac{{\left[K\right]}_{o}}{{\left[K\right]}_{i}}\right)-\ln\left(\frac{{\left[\hat{K}\right]}_{o}}{{\left[\hat{K}\right]}_{i}}\right)\right]\\ =\frac{RT}{F}\left[\ln\left(\frac{{\left[K\right]}_{o}}{{\left[\hat{K}\right]}_{o}}\right)-\ln\left(\frac{{\left[K\right]}_{i}}{{\left[\hat{K}\right]}_{i}}\right)\right]\\ \approx \frac{RT}{F}\ln\left(\frac{{\left[K\right]}_{o}}{{\left[\hat{K}\right]}_{o}}\right) \end{array}$$ (1)

Here $\frac{RT}{F}=\frac{k_{b}T}{e}\approx 26.64$ . [**.**]_*o*_ and [**.**]_*i*_ represent extracellular and intracellular concentrations, respectively. The nominal values for Nernst potentials for excitatory and inhibitory masses are −75 mV and −90 mV, respectively. For these ${\hat{\nu }}_{K}$ s and chosen [*K*]_*i*_ of 140 mM, this gives nominal extracellular concentrations as 4.77 mM and 8.38 mM, respectively. Because, the intracellular volume fraction is much larger than the extracellular volume fraction, and the intracellular potassium concentration is much larger than the extracellular concentration, the fractional changes in intracellular concentration is generally negligible. Hence, Δ*ν*_*K*_ is dominantly due to fractional changes in extracellular potassium concentration, as expressed by last line of Eq. 1.

**FIGURE 2:**
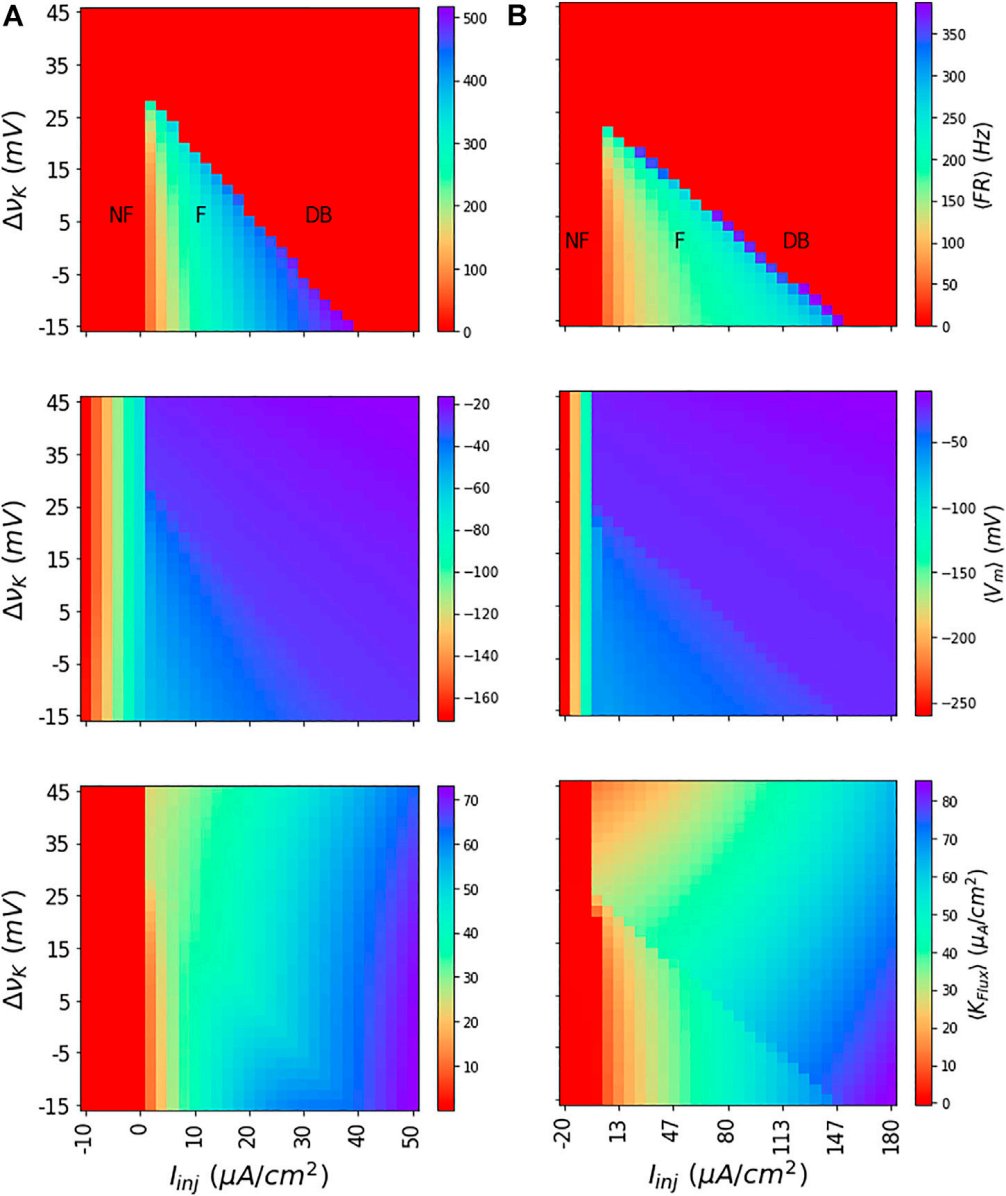
Dynamics of WB and SEAN models: Shown for the WB (A) and SEAN (B) models are the mean firing rate ⟨FR⟩ (top row); mean membrane potential ⟨*V*_*m*_⟩ (middle row), and mean potassium flux ⟨*K*_*FLux*_⟩ (bottom row). For the red portions of the colour maps in the first row (⟨FR⟩ = 0), the models are either not firing (NF) or in DB. The difference in firing (F) and DB regions can be understood in terms of ⟨*V*_*m*_⟩, and ⟨*K*_*FLux*_⟩ colour maps. While the NF region shows very low negative values for ⟨*V*_*m*_⟩, the DB region has them at higher negative values. Similarly, in NF region, the ⟨*K*_*FLux*_⟩ is either negative or zero, in the DB region it is finite and always positive. The nominal values of the Nernst potentials correspond to Δ*ν*_*K*_ = 0 for both the models, and these are *ν*_*K*_ = −90 *mV* for the WB model, and *ν*_*K*_ = −75 *mV* for the SEAN model.

Given a Hodgkin-Huxley type single cell neuron model (i.e., the Single-compartment Excitatory Accommodating Neuron (SEAN) and Wang- Buzsáki (WB) models, described next), the procedure for developing the corresponding mNMs is:

**Identify the dynamical regimes of interest.** We obtain the dynamics of the neuron model at different values of injected currents (*I*_inj_) and *ν*_*K*_ to obtain membrane potential traces. Following this, we find the corresponding mean firing rate (⟨FR⟩), and mean membrane potential (⟨*V*_*m*_⟩) and mean potassium flux (⟨*K*_*flux*_⟩) for each values of *I*_inj_ and *ν*_*K*_ by averaging the dynamics. As shown in Figure 2 for both the WB and the SEAN models for discrete choices of *I*_inj_ and *ν*_*K*_ values, this subdivides (*I*_inj_, *ν*_*K*_) parameter space into regions of not-firing (NF), firing (F), and depolarization block (DB).

The depolarization block region that occurs at higher values of *I*_inj_ and *ν*_*K*_ is characteristically different from not-firing region that occurs at low *I*_inj_ values. While the NF region has either negative or zero ⟨*K*_*flux*_⟩, the DB region has finite and positive ⟨*K*_*flux*_⟩. Similarly, the ⟨*V*_*m*_⟩ shows very negative values in the NF region, whereas it shows higher values in the DB region.

**Identification of bifurcation boundaries.** After identification of the three dynamical regimes of interest, we then analytically identify the bifurcation boundaries.

**FIGURE 3:**
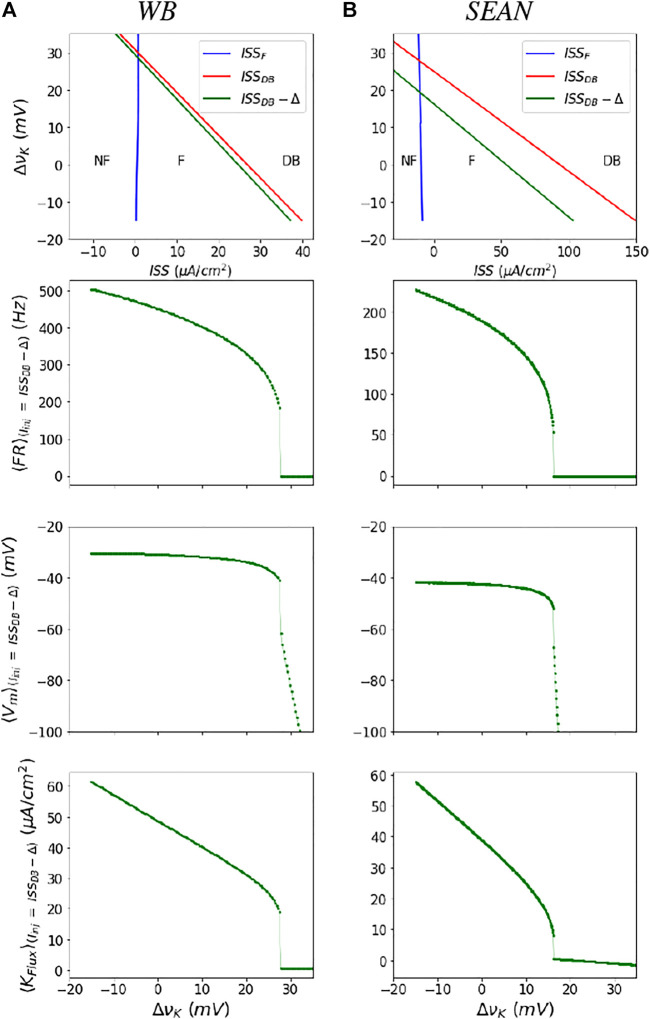
Parametrization of the neural masses for the WB **(A)** and SEAN **(B)** models: In the top row are shown the boundaries of the firing (F) region in terms of steady state current (ISS) and change in potassium Nernst potential $\Delta \nu_{K}$ . The blue and red boundaries (ISSF and ISSDB, respectively) are obtained from the steady state analysis of the WB model (refer to the supplementary). We parametrize the maximal firing rate, and related outputs along the green line (ISSDB − Δ). Shown in the subsequent rows are the mean firing rate <*FR*>, mean transmembrane potential <*V_m_* >, and mean potassium flux <*K_flux_* >, as a function $\Delta \nu_{K}$ along the boundary at (ISSDB − Δ) (closed symbols), along with the polynomial fits (solid lines), used to parameterize the masses. The nominal values of the Nernst potentials correspond to $\Delta \nu_{K}=0$ for both the models. The nominal values are $\Delta \nu_{K}=-90$ mV for the WB model, and $\Delta \nu_{K}=75$ mV for the SEAN model.

For the WB and SEAN models, we identify the F and DB thresholds in *I*_inj_ and *ν*_*K*_ state space. For this, we find stability of the fixed points of the models using eigenvalues of the model Jacobian. In practice the fixed points in these models are more easily solved as functions of *V*_*m*_. The point where the largest eigenvalue crosses zero marks the transitions between stable and unstable regions. The two boundaries of the unstable triangular region are shown as blue (ISS_*F*_) and red (ISS_*DB*_) lines in top row of Figure 3 for both the models. The boundaries are the steady state currents at F onset and DB onset. We can observe from this figure that for each *ν*_*K*_ (also each row in the heat map in Figure 2), there are two values of currents that mark the firing (unstable) region. At the lower of these current values the ⟨*FR*⟩ is zero and at the higher values the ⟨*FR*⟩ is at its maximum value, for that *ν*_*K*_.

Note that while the dynamics in the lower triangular region bounded by the *ISS*_*F*_ and *ISS*_*DB*_ lines is characterized by unstable fixed point dynamics, the upper triangular regions (at high Δ*ν*_*K*_ are characterized by two stable fixed points - one that is NF and the other in DB.

**Find mean dynamical quantities near bifurcation boundaries.** After having obtained the thresholds, we find the mean of dynamical variables (⟨*FR*⟩, ⟨*V*_*m*_⟩, and ⟨*K*_*flux*_⟩) near the thresholds (ISS_*F*_ and ISS_*DB*_). We use these means to parameterize these quantities within the enclosed firing region. Outside these regions the means are analytically defined by the stable fixed point solutions.

The mean firing rate is a smooth, nearly square-root shaped function of ISS, except near the DB boundary (refer Supplementary Figure S1), where it increases sharply and the models produce incomplete action potentials. Therefore, in practice, we compute these averages on a line that is slightly shifted to (ISS_*DB*_(*ν*_*K*_) − Δ), as shown in green in Figure 3. Moreover, by using this left shifted boundary, we avoid taking very noisy and high firing rates with incomplete action potentials that likely do not produce synaptic transmission to postsynaptic cells. Averages quantities along the green boundary, for a range of changes in *ν*_*K*_, for these models are shown in next three rows of Figure 3. We treat the ⟨*FR*⟩ and other mean dynamical quantities at ISS_*DB*_ − Δ as their “maximum” values in the firing region before the model goes into DB.

**Neural Mass parametrization: Finding functional fits to time-averaged dynamical quantities.** After obtaining the fairly smooth boundaries of unstable region and mean dynamical quantities as a function of Δ*ν*_*K*_, we find and polynomial functions that parameterize the bifurcation boundaries ISS_*F*_(*ν*_*k*_) and ISS_*DB*_, as well as ⟨*FR*⟩, ⟨*V*_*m*_⟩, and ⟨*K*_*flux*_ (*ν*_*k*_)⟩ along these boundaries. So, for any given *ν*_*k*_, these analytical functions would give the F, DB thresholds, and “maximum” values of the dynamical quantities. These functions are then used to parametrize the dynamical quantities between the boundaries, which appear with nearly square-root behavior. These resulting equations form our mNM behavior.
**Check Fidelity of neural mass parametrization.** Within the firing region, defined by the parametrized boundaries $ISS_{F}\left(\Delta \nu_{K}\right)$ and $ISS_{DB}\left(\Delta \nu_{K}\right)$ , we find that the neural mass outputs FR , $V_{m}$ , and $K_{flux}$ , are well approximated by square root functions of the normalized injected current times the parametrized maximal value of the quantity found along the DB boundary. To check fidelity of this parameterization, we then compare the neural mass outputs with the means of the same quantities obtained from the ordinary differential Equation (ODE) simulations of the respective neuron models to ascertain if the mNM outputs are in the reasonable limits of the error.

### 2.2 The Wang-Buzsáki and Single-Compartment Excitatory Accommodating Neuron Neuron Models Used

In the next two sections we give the ODE equations and details of the WB and SEAN neuron models that we use to obtain the I and E neural masses.

#### 2.2.1 Wang-Buzsa´ki Model for Inhibitory Neuron

The model equations of the Wang-Buzsáki (Wang and Buzsáki, 1996) neuron are as follows:

$$\begin{array}{l} \frac{dV_{m}}{dt}=\frac{1}{C_{m}}\left(I_{\mathrm{inj}}-G_{K}\left(V_{m}-\nu_{K}\right)-G_{Na}\left(V_{m}-\nu_{Na}\right)-G_{L}\left(V_{m}-\nu_{l}\right)-I_{\mathrm{pump}}\right)\\ \frac{dn}{dt}=\psi \left(\alpha_{n}\left(V_{m}\right)\left(1-n\right)-n\beta_{n}\left(V_{m}\right)\right)\\ \frac{dh}{dt}=\psi \left(\alpha_{h}\left(V_{m}\right)\left(1-h\right)-h\beta_{h}\left(V_{m}\right)\right) \end{array}$$ (2)

where,

$$\begin{array}{ll} G_{K} & =g_{k}n^{4}\\ G_{Na} & =g_{Na}m_{\infty }^{3}h\\ G_{L} & =g_{L}\\ I_{pump} & =\frac{\rho }{\left(1+\exp\left(\frac{{\left[\hat{N}a\right]}_{i}-{\left[Na\right]}_{i}}{3}\right)\right)\left(1+\exp\left({\left[\hat{K}\right]}_{o}-{\left[K\right]}_{o}\right)\right)} \end{array}$$ (3)

The expression for the pump current and the value of pump strength *ρ* = 1.25 is taken from (Wei et al., 2014). Nominal ion concentrations for intracellular sodium and extracellular potassium are ${\left[\hat{N}a\right]}_{i}=25\;mM$ , ${\left[\hat{K}\right]}_{o}=4.77\;mM$ , and the nominal Nernst potentials for these ions are ${\hat{\nu }}_{K}=-90\;mV,\;{\hat{\nu }}_{Na}=55\;mV$ . The Nernst potential for sodium ion is assumed to be constant, i.e., $\nu_{Na}={\hat{\nu }}_{Na}$ . Non-hatted versions of concentrations incorporate the changes in Nernst potentials. In this work, we assume that the intracellular concentration remain constant at all times, hence in the pump current (*I*_*pump*_), the changes in *ν*_*K*_ is fully accounted by [*K*]_*o*_.

The activation functions for the ion channels are given by the following equations:

$$\begin{array}{l} \alpha_{m}\left(V_{m}\right)=\frac{a_{1_{W}}\left(V_{m}+V_{1_{W}}\right)}{\left(1-\exp\left(-\left(V_{m}+V_{1_{W}}\right)/b_{1_{W}}\right)\right)}\\ \beta_{m}\left(V_{m}\right)=a_{2_{W}}\exp\left(-\left(V_{m}+V_{2_{W}}\right)/b_{2_{W}}\right)\\ \alpha_{n}\left(V_{m}\right)=\frac{a_{3_{W}}\left(V_{m}+V_{3_{W}}\right)}{\left(1-\exp\left(-\left(V_{m}+V_{3_{W}}\right)/b_{3_{W}}\right)\right)}\\ \beta_{n}\left(V_{m}\right)=a_{4_{W}}\exp\left(-\left(V_{m}+V_{4_{W}}\right)/b_{4_{W}}\right)\\ \alpha_{h}\left(V_{m}\right)=a_{5_{W}}\exp\left(-\left(V_{m}+V_{5_{W}}\right)/b_{5_{W}}\right)\\ \beta_{h}\left(V_{m}\right)=\frac{1}{\left(1+\exp\left(-\left(V_{m}+V_{6_{W}}\right)/b_{6_{W}}\right)\right)} \end{array}$$ (4)

The values of the model parameters are given in the first column of Supplementary Table S1. The mean outputs of the model obtained from the ODE simulations are depicted as colour maps in Figure 2A. The ⟨*K*_*Flux*_⟩ current has contributions from active potassium current (*G*_*K*_(*V*_*m*_ − *ν*_*K*_)) and the pump current.

The fixed point of the system of WB model equations for a given membrane potential are given by (*V*_*m*_, *n*_*∞*_(*V*_*m*_), *m*_*∞*_(*V*_*m*_), *h*_*∞*_(*V*_*m*_)), and its stability is computed from the Jacobian of the dynamics at that point. At the fixed point, the input current *I*_*SS*_ for a given *V*_*m*_ and *ν*_*K*_ is given by:

$$I_{SS}=g_{K}n_{\infty }^{4}\left(V_{m}\right)\left(V_{m}-\nu_{K}\right)+g_{Na}m_{\infty }^{3}\left(V_{m}\right)h_{\infty }\left(V_{m}\right)\left(V_{m}-V_{Na}\right)+g_{L}\left(V_{m}-V_{l}\right)+I_{\mathrm{pump}}$$ (5)

The boundaries of the unstable region in terms of the Firing boundary (ISS_*F*_) and Depolarization block boundary (ISS_*DB*_) are calculated using the above expression and depicted in the left column of Figure 3.

#### 2.2.2 Single-Compartment Excitatory Accommodating Neuron Model

We obtain the SEAN model from the Pinksy-Rinzel model (Pinsky and Rinzel, 1994) by converting it to a single-compartment model and retaining major active currents along with the accommodation (*I*_*K*−*AHP*_) and the pump current (*I*_*pump*_).

The equations of the SEAN model are as follows:

$$\begin{array}{l} \frac{dV_{m}}{dt}=\frac{1}{C_{m}}\left(I_{\mathrm{inj}}-G_{K}\left(V_{m}-\nu_{K}\right)-G_{Na}\left(V_{m}-\nu_{Na}\right)-G_{L}\left(V_{m}-\nu_{l}\right)-I_{\mathrm{pump}}-I_{K-AHP}\right)\\ \frac{dn}{dt}=\alpha_{n}\left(V_{m}\right)\left(1-n\right)-n\beta_{n}\left(V_{m}\right)\\ \frac{dh}{dt}=\alpha_{h}\left(V_{m}\right)\left(1-h\right)-h\beta_{h}\left(V_{m}\right) \end{array}$$ (6)

where,

$$\begin{array}{ll} G_{K} & =g_{k}n^{4}\\ G_{Na} & =g_{Na}m_{\infty }^{3}h\\ G_{L} & =g_{L}\\ I_{pump} & =\frac{\rho }{\left(1+\exp\left(\frac{{\left[\hat{N}a\right]}_{i}-{\left[Na\right]}_{i}}{3}\right)\right)\left(1+\exp\left({\left[\hat{K}\right]}_{o}-{\left[K\right]}_{o}\right)\right)}\\ I_{K-AHP} & =G_{K-AHP}q_{\infty }\left(Ca\right)\left(V_{m}-\nu_{K}\right) \end{array}$$ (7)

where *ρ* = 1.25 is the pump strength. ${\left[\hat{N}a\right]}_{i}=25$ mM, ${\left[\hat{K}\right]}_{o}=8.38$ mM stand for intracellular sodium and extracellular potassium concentrations for nominal Nernst potentials for these ions (*ν*_*K*_ = −75 mV, *ν*_*Na*_ = 60 mV). Non-hatted versions of these quantities incorporate the changes in Nernst potentials.

The activation variables of different ions as a function *V*_*m*_ are:

$$\begin{array}{l} \alpha_{m}\left(V_{m}\right)=\frac{-a_{1_{S}}\left(V_{1_{S}}+V_{m}\right)}{\left(\exp\left(-\left(V_{1_{S}}+V_{m}\right)/b_{1_{S}}\right)-1.0\right)}\\ \beta_{m}\left(V_{m}\right)=\frac{a_{2_{S}}\left(V_{m}+V_{2_{S}}\right)}{\left(\exp\left(\left(V_{m}+V_{2_{S}}\right)/b_{2_{S}}\right)-1.0\right)}\\ \alpha_{n}\left(V_{m}\right)=\frac{-a_{3_{S}}\left(V_{3_{S}}+V_{m}\right)}{\left(\exp\left(-\left(V_{3_{S}}+v\right)/b_{3_{S}}\right)-1.0\right)}\\ \beta_{n}\left(V_{m}\right)=a_{4_{S}}\exp\left(-\left(V_{4_{S}}+V_{m}\right)/b_{4_{S}}\right)\\ \alpha_{h}\left(V_{m}\right)=a_{5_{S}}\exp\left(-\left(V_{5_{S}}+V_{m}\right)/b_{5_{S}}\right)\\ \beta_{h}\left(V_{m}\right)=\frac{a_{6_{S}}}{\left(1.0+\exp\left(-\left(V_{6_{S}}+V_{m}\right)/b_{6_{S}}\right)\right)} \end{array}$$ (8)

The potassium after-hyper-polarization current *I*_*K*−*AHP*_ is the current responsible for accommodation in this model and calcium activation variable *q* is assumed to be at its steady value:

$$q_{\infty }\left(Ca\right)=\frac{\alpha_{q}}{\left(\alpha_{q}+\beta_{q}\right)}$$ (9)

where *α*_*q*_ = min (0.00002∗*Ca*, 0.01) and *β*_*q*_ = 0.001.

As in (Pinsky and Rinzel, 1994), the calcium dynamics themselves evolve relatively slowly according to:

$$\frac{dCa}{dt}=-0.13I_{Ca}-0.075Ca$$ (10)

We then make the follow approximation to separate the effects of the *I*_*K*−*AHP*_ into fast dynamical (*I*_*K*−*AHP*,*fast*_) effects computed at the nominal state and slow modulatory effects that depend on calcium (*I*_*K*−*AHP*,*slow*_ (*Ca*)):

$$\begin{array}{l} I_{K-AHP}=g_{K-AHP}\left(\hat{q}+q_{\infty }\left(Ca\right)-\hat{q}\right)\left(V_{m}-\nu_{K}\right)\\ =g_{K-AHP}\hat{q}\left(V_{m}-\nu_{K}\right)+g_{K-AHP}\left(q_{\infty }\left(Ca\right)-\hat{q}\right)\left(V_{m}-\nu_{K}\right)\\ \approx g_{K-AHP}\hat{q}\left(V_{m}-\nu_{K}\right)+\langle g_{K-AHP}\left(q_{\infty }\left(Ca\right)-\hat{q}\right)\left(V_{m}-\nu_{K}\right)\rangle \\ \approx g_{K-AHP}\hat{q}\left(V_{m}-\nu_{K}\right)+g_{K-AHP}\left(q_{\infty }\left(Ca\right)-\hat{q}\right)\left(\langle V_{m}\rangle -\nu_{K}\right)\\ =I_{K-AHP,fast}+I_{K-AHP,slow}\left(Ca\right) \end{array}$$ (11)

In practice, the mNM parametrization is computed just using the fast dynamics *I*_*K*−*AHP*,*fast*_, where we have chosen $\hat{q}=q_{\infty }\left(Ca=0.2\right)$ . The slow dynamics appear as an additive correction to the input current *I*_*inj,eff*_ = *I*_inj_ + *I*_*K*−*AHP*,*slow*_.

The values of the model parameters are given in the second column of Supplementary Table S1. The mean outputs of the model obtained from the ODE simulations are depicted in Figure 2B. The ⟨*K*_*Flux*_⟩ current has contributions from the active potassium current (*G*_*K*_(*V*_*m*_ − *ν*_*K*_)), the pump current, and the accommodation current.

The model fixed points are determined similarly as the previous model. The steady state value of the current at given (*V*_*m*_ and *ν*_*K*_) are determined as:

$$I_{SS}=g_{K}n_{\infty }\left(V_{m}\right)\left(V_{m}-\nu_{K}\right)+g_{Na}m_{\infty }^{3}\left(V_{m}\right)h_{\infty }\left(V_{m}\right)\left(V_{m}-V_{Na}\right)+g_{L}\left(V_{m}-V_{l}\right)+I_{\mathrm{pump}}+I_{K-AHP}$$ (12)

The firing onset (ISS_*F*_) and DB (ISS_*DB*_) boundaries in terms of steady state current are depicted in Figure 3.

### 2.3 Mechanistic Neural Mass Parametrization

We use the functional fits to the firing and DB thresholds, and the mean of dynamical variables at (adjusted) DB threshold (ISS_*DB*_ − Δ) to parameterize the mNMs. For both the neuron models, we use polynomial functions up to 3rd order to fit these quantities as a function of *ν*_*K*_ (*x*). For instance, for functional fit to ISS_*F*_ of WB model, we obtain the best linear fit (*y* = *ax* + *b*) with *a* = 0.014, *b* = 1.746 as parameters. All the functional best fits for both the models are shown in Supplementary Figures S2 and S3. The order of polynomial was simply chosen so as to fit the profile of the simulated data (see Supplementary Figures S2 and S3), and python’s Scipy (docs.scipy.org) module was used to obtain the best fit. These functions are used in ascertaining the limits of the three dynamical quantities, for a given *ν*_*K*_.

As a next step we separately identify another set of simple functions that would approximate the values of the dynamical variables of the mass, within the predetermined limit, as a function of the injected current. These functions (example $y=M\sqrt{x}+C$ ) use the input current (injected current) as independent variable (x) and the predetermined limits of dynamical variables (*M*) to yield mean dynamical output (*y*) in terms of mean firing rate, mean membrane potential, or, mean potassium flux.

In summary, for any given combination of (I_inj_, *ν*_*K*_), we first determine the threshold currents, and maximum values of mean dynamical variables from the first set of analytical functions. Next, we use the I_inj_, as the independent variable in the next set of functions and find the dynamical variables corresponding to the mass. The choice of these functions was guided by the profiles of average firing rate ⟨*FR*⟩, membrane potential ⟨*V*_*m*_⟩, and potassium flux ⟨*K*_*flux*_⟩ of individual neuron models as function of I_inj_. These are shown for three different *ν*_*K*_ values for both the neuron models in Supplementary Figure S1.

After the neural masses are characterized, we check their fidelity by comparing the mass outputs with that of mean outputs from the ODE simulations of the neuron models across the ranges of *I*_inj_ and *ν*_*K*_.

#### 2.3.1 Determination of Thresholds for a Given Change in the Nernst Potential

The thresholds current values and maximum of the means of FR, V_*m*_, and K_*Flux*_ within the firing range are determined using simple linear and polynomial functions of *ν*_*K*_ of the two masses. Hence, firstly, for a given change in the potassium Nernst potential Δ*ν*_*K*_, the corresponding (new) Nernst potentials of the two masses are:

$$\nu_{K_{I}}={\hat{\nu }}_{K_{I}}+\Delta \nu_{K}$$ (13a)

$$\nu_{K_{E}}={\hat{\nu }}_{K_{E}}+\Delta \nu_{K}$$ (13b)

The hatted versions of the Nernst potentials ( ${\hat{\nu }}_{K_{I}}$ and ${\hat{\nu }}_{K_{E}}$ ) are the nominal Nernst potential values of the two masses. Note that amount of change in Nernst potential is assumed to be caused only due to change in extracellular potassium concentrations for the two masses, and these masses share a common extracellular bath (see Figure 1). Hence Δ*ν*_*K*_ is same for both the masses.

The functions that determine the firing threshold $\left(i_{I_{\mathrm{th1}}}\right)$ , depolarization threshold $\left(i_{I_{\mathrm{th2}}}\right)$ , maximum $\left\langle \text{FR}\right\rangle \;\left(M_{1}^{I}\right)$ , maximum $\left\langle V_{m}\right\rangle \;\left(M_{2}^{I}\right)$ , and maximum $\left\langle K_{\mathrm{Flux}}\right\rangle \;\left(M_{3}^{I}\right)$ , as functions of $\nu_{K_{I}}$ for the I mass are:

$$i_{I_{\mathrm{th1}}}=a_{I_{\mathrm{FRT}}}\nu_{K_{I}}+b_{I_{\mathrm{FRT}}}$$ (14a)

$$i_{I_{\mathrm{th2}}}=a_{I_{\mathrm{DBT}}}\nu_{K_{I}}+b_{I_{\mathrm{DBT}}}$$ (14b)

$$M_{1}^{I}=a_{I_{FR}}\nu_{K_{I}}^{3}+b_{I_{FR}}\nu_{K_{I}}^{2}+c_{I_{FR}}\nu_{K_{I}}+d_{I_{FR}}$$ (14c)

$$M_{2}^{I}=a_{I_{V_{m}}}\nu_{K_{I}}^{2}+b_{I_{V_{m}}}\nu_{K_{I}}+c_{I_{V_{m}}}$$ (14d)

$$M_{3}^{I}=a_{I_{KF}}\nu_{K_{I}}+b_{I_{KF}}$$ (14e)

Similarly, the firing threshold $\left(i_{E_{\mathrm{th1}}}\right)$ , depolarization threshold $\left(i_{E_{\mathrm{th2}}}\right)$ , maximum $\left\langle V_{m}\right\rangle \;\left(M_{1}^{E}\right)$ , maximum $\left\langle V_{m}\right\rangle \;\left(M_{2}^{E}\right)$ , and maximum $\left\langle K_{\mathrm{Flux}}\right\rangle \;\left(M_{3}^{E}\right)$ functions (of $\nu_{K_{E}}$ ) that determine the threshold values for the E mass are:

$$i_{E_{\mathrm{th1}}}=a_{E_{\mathrm{FRT}}}\nu_{K_{E}}+b_{E_{\mathrm{FRT}}}$$ (15a)

$$i_{E_{\mathrm{th2}}}=a_{E_{\mathrm{DBT}}}\nu_{K_{E}}+b_{E_{\mathrm{DBT}}}$$ (15b)

$$M_{1}^{E}=a_{E_{FR}}\nu_{K_{E}}^{2}+b_{E_{FR}}\nu_{K_{E}}+c_{E_{FR}}$$ (15c)

$$M_{2}^{E}=a_{E_{V_{m}}}\nu_{K_{E}}^{2}+b_{E_{V_{m}}}\nu_{K_{E}}+c_{E_{V_{m}}}$$ (15d)

$$M_{3}^{E}=a_{E_{KF}}\nu_{K_{E}}^{3}+b_{E_{KF}}\nu_{K_{E}}^{2}+c_{E_{KF}}$$ (15e)

The values of the parameters appearing in the above equations Eqs. 14 and 15 are presented in Supplementary Table S2. The functions are plotted against actual thresholds for a range of *ν*_*K*_ values and shown in Supplementary Figures S1 and S2, for the I and the E mass, respectively, in the SI.

#### 2.3.2 Neural Mass Functionals

The parameters of the above linear and polynomial functions can be used to determine these thresholds for any arbitrary Δ*ν*_*K*_. These thresholds are further used in another set of functions to determine the mean dynamical outputs of the mass for any given injected current. As these functions fully characterize the neural mass with respect to the bifurcation parameters (*I*_inj_ and *ν*_*K*_), we address them as the neural mass functionals. We present these functionals below for the I and the E masses.

The mean firing rate function (FR_*I*_), mean membrane potential function $\left(V_{m_{I}}\right)$ , mean potassium flux functions (*KF*_*I*_) for the I mass are:

$$FR_{I}=M_{1}^{I}\sqrt{Q_{I}}$$ (16a)

$$V_{m_{I}}=-M_{2}^{I}\left(\sqrt{Q_{I}}\right)+C1$$ (16b)

$$KF_{I}=M_{3}^{I}\left(\sqrt{Q_{I}}\right)$$ (16c)

where, *C*1 = −60, $M_{1}^{I}$ , $M_{2}^{I}$ , and $M_{3}^{I}$ are the maximum of the respective quantities as determined for a given $\nu_{K_{I}}$ from Eq. 14. *Q*_*I*_ is the normalized total current that the mass receives and is expressed as:

$$Q_{I}=\frac{X_{I_{\mathrm{tot}}}-i_{I_{\mathrm{th1}}}}{i_{I_{\mathrm{th2}}}-i_{I_{\mathrm{th1}}}}$$ (17)

Notice that, for the isolated mass this total current $\left(X_{I_{\mathrm{tot}}}\right)$ is just the injected current $\left(I_{I_{\mathrm{inj}}}\right)$ . Alternatively, when this mass is coupled to other mass (es), the total current is the sum of injected current and the current due to finite ⟨FR⟩ of other mass (es). Hence, the expression for the total current for the mass (U), that couples to another mass (V) is:

$$X_{U_{\mathrm{tot}}}=I_{U_{\mathrm{inj}}}+I_{U_{\mathrm{pump}}}\left(\Delta \nu_{K}\right)+g_{VU}FR_{V}$$ (18)

The mean firing rate function (*FR*_*E*_), mean membrane potential function $\left(V_{m_{E}}\right)$ , mean potassium flux functions (*KF*_*E*_) for the E mass are:

$$FR_{E}=M_{1}^{E}\sqrt{Q_{E}}$$ (19a)

$$V_{m_{E}}=-M_{2}^{E}\left(Q_{E}^{0.62}\right)+C1$$ (19b)

$$KF_{E}=M_{3}^{E}\left(Q_{E}^{0.28}\right)+C2$$ (19c)

where, *C*1 = −65 and *C*2 = 0.1 and normalized total current (*Q*_*E*_) is expressed as:

$$Q_{E}=\frac{X_{E_{\mathrm{tot}}}-i_{E_{\mathrm{th1}}}}{i_{E_{\mathrm{th2}}}-i_{E_{\mathrm{th1}}}}$$ (20)

Here again, the total current $X_{E_{\mathrm{tot}}}$ can have up to three contributions as expressed in Eq. 18.

### 2.4 Synaptic Coupling of Masses

So far, we have presented how we develop the neural masses of both kinds (E and I) from their respective neuron models. Next, we would like to understand how does the coupled E − I model, in which the two masses interact with each other through synaptic coupling, behaves under the conditions of changing injected currents and changes in the *ν*_*K*_ of both the masses.

Mass coupling can also be understood from an averaging approach from classic Hodgkin-Huxley (HH) type compartment models. We write this out both for completeness, and to illustrate a slight change from existing NMMs.

In the HH like models originally input is only in the form of current. In neuronal networks, this is mediated by synaptic currents, whose impulse-response function in response to neurotransmitter release at time *t*_*ap*_ has been characterized by alpha functions *α*(*t*) (Rall et al., 1967; Brown and Johnston, 1983; Traub and Miles, 1991).

$$\begin{array}{l} I\left(t-t_{ap}\right)=G\alpha \left(t\right)\left(V_{m}-\nu_{s}\right)\\ \alpha \left(t\right)=te^{-t/\tau_{s}} \end{array}$$ (21)

Note that the time constant *τ*_*s*_ is particular to a type of neurotransmitter, and we will in the future denote as a generalization *α*_*x*_(*t*) to have time constant *τ*_*x*_. Note that the term *V*_*m*_ − *ν*_*s*_ is the difference between membrane potential *V*_*m*_ and the Nernst-potential of carrier ions through the channel of type *s*. Commonly used time constants include *τ*_*p*_ = 10 ms for pyramidal neurons, *τ*_*I*,*f*_ = 2 ms for fast (*γ*-aminobutyric acid GABA_*A*_) inhibitory, and *τ*_*I*,*s*_ = 20 ms for slow (GABA_*B*_) inhibitory interneurons (Traub and Miles, 1991).

For a single neuron, denoted as neuron *i*, with firing times at its soma of *T*_*k*_, its synapses onto neurons will occur at axonal terminals, and there will be a delay time *D* mostly proportional to the distance such that action potentials will be seen at the synapse at times *T*_*i*,*k*_ + *D*_*i*,*b*_. Critically, delay time *D*_*i*,*b*_ is specific to the connection distance between neurons *i* and *b*.

We note that a particular neuron type, such as pyramidal neuron, will only release a single neurotransmitter type. Then the time course of postsynaptic currents from firing of that neuron to postsynaptic neuron *b*, will have the form:

$$I_{b}\left(t\right)=G_{i,b}\left(V_{m,b}-\nu_{b}\right)\underset{k}{\sum }\alpha_{i}\left(t-T_{i,k}-D_{i,b}\right)$$ (22)

It is now convenient to introduce a pre-synaptic centric conductance function *h*_*a*,*i*_(*t*) for the *i*th single cell from population *a*, assumed to be all of the same cell type:

$$h_{a,i}\left(t\right)\equiv \underset{k}{\sum }\alpha_{a,i}\left(t-T_{a,k}\right)$$ (23)

In this form, any postsynaptic current in neuron *b* from action potentials in *i* will be characterized as,

$$I_{b}\left(t\right)=G_{a,b}\left(V_{m,b}-\nu_{b}\right)h_{a,i}\left(t-D_{a,b}\right)$$ (24)

In our neural mass formalism, we now average over a population *a*, composed of neurons *i*, all with the same neurotrasmitter type, and assumed to have common time delay *D*_*a*,*b*_. As such *α*_*a*,*i*_ = *α*_*a*_. As a result we arrive at an equivalent averaged conductance function *h*_*a*_(*t*),

$$h_{a}\left(t\right)={\left\langle \underset{i,k}{\sum }\alpha_{a,i}\left(t-T_{i,k}\right)\right\rangle }_{i,k}$$ (25a)

$$={\left\langle \underset{i,k}{\sum }\int d\tau \alpha_{a}\left(t-\tau \right)\delta \left(\tau -T_{i,k}\right)\right\rangle }_{i,k}$$ (25b)

$$=\int d\tau \alpha_{a}\left(t-\tau \right){\left\langle \underset{i,k}{\sum }\delta \left(\tau -T_{i,k}\right)\right\rangle }_{i,k}$$ (25c)

$$=\int d\tau \alpha_{a}\left(t-\tau \right)F_{a}\left(\tau \right)$$ (25d)

Here we’ve defined the firing rate for the neuron group defined by *a* as *F*_*a*_(*τ*).

It can be shown that *h*_*a*_(*t*) can be computed without the time convolution by instead integrating the second order ordinary differential equation:

$${\ddot{h}}_{a}=\frac{F_{a}-h_{a}}{\tau_{a}^{2}}-\frac{2{\dot{h}}_{a}}{\tau_{a}}$$ (26)

Note that under this parametrization, under steady state conditions *F*_*a*_(*t*) = *F*_*a*_ and ${\dot{h}}_{a}=0$ then *h*_*a*_ = *F*_*a*_.

For computational efficiency, for masses that are coupled into networks with both near and far connections, we only compute the synaptic dynamics once per mass, and accommodate the delays in their connections. Explicitly, for NM for population *b*, whose presynaptic masses are denoted as members *a*, will have a total input current

$$I_{b,in}\left(t\right)=I_{b,pump}+\underset{a}{\sum }G_{a,b}\left(\left\langle V_{m}\right\rangle -\nu_{b}\right)h_{a}\left(t-D_{a,b}\right)$$ (27)

and idealized instantaneous firing rate defined by our parametrization *f*_*b*_ (*I*_*b*,*in*_(*t*), *θ*_*b*_(*t*)).

Note that because synaptic current is slow with respect to the fast dynamics, and appears after averaging across the post-synaptic network, the term *V*_*m*_ in Eq. 21 is replaced with the NM state ⟨*V*_*m*_⟩. This is commonly further replaced with a nominal value (as in (Deschle et al., 2021)) ${\hat{V}}_{m,b}$ . This may be rationalized by assumption that the synapses, which typically appear distant from the neural Soma modeled, are not well-described by the mean Soma membrane potential. In the latter case the term $G_{a,b}\left({\hat{V}}_{m,b}-\nu_{b}\right)$ is constant, and replaced with an overall coupling constant *g*_*a*,*b*_.

$$I_{b,in}\left(t\right)=I_{b,pump}+\underset{a}{\sum }g_{a,b}h_{a}\left(t-D_{a,b}\right)$$ (28)

## 3 Results

As described, we have developed mNMs as parameterizations of single-neuron Hodgkin-Huxley style neurons for both inhibitory and excitatory neuron types, based on the WB and SEAN models. As results, we first demonstrate that the individual mNMs parametrizations reproduce the outputs of the demonstrated models.

We then investigate the dynamics of the simple 2-element excitatory/inhibitory network illustrated in Figure 1 and demonstrate that as a function of both input and extracellular potassium, it reveals dynamics expected to underlie seizure and spreading depression dynamics.

### 3.1 Mechanistic Neural Mass Fidelity

**FIGURE 4:**
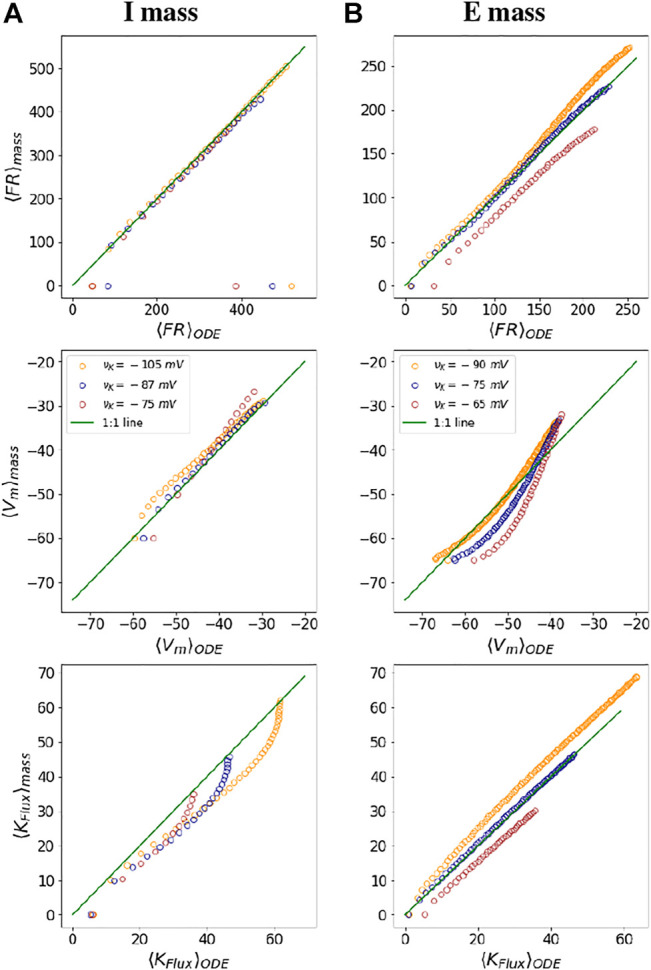
Fidelity of mass parametrization: The figures show a comparison of mean dynamical variables obtained from ODE simulations with those obtained from neural mass parametrization at three different values of *ν*_*K*_ for the *I* mass **(A)** and the *E* mass **(B)**, respectively. The green line in all the plots depicts a 1:1 line for the ODE and neural mass outputs. The colours of the dots in the figure are indicative of different potassium Nernst potential values, as depicted in the middle subplots. The points parallel the *x* axis in the subplots at firing onset and at DB onset, depict areas where the mass model is not exactly approximating the mean outputs from the neuron models.

To check the fidelity of the mechanistic neural mass parametrization, we compare the outputs obtained from the I and E neural functionals with that of mean outputs from the WB and SEAN neuron ODE models. For both the neurons, we begin by choosing a range of injected currents for which the neurons fire and obtain the mean quantities (⟨*FR*⟩_*ODE*_, ${\left\langle V_{m}\right\rangle }_{\mathrm{ODE}}$ , and ${\left\langle K_{\mathrm{Flux}}\right\rangle }_{\mathrm{ODE}}$ ) at three different *ν*_*K*_ values. We also find out these three quantities (⟨*FR*⟩_*mass*_, ${\left\langle V_{m}\right\rangle }_{\mathrm{mass}}$ , and ${\left\langle K_{\mathrm{Flux}}\right\rangle }_{\mathrm{mass}}$ ), using the neural mass functionals for the same range of injected currents and for the same *ν*_*K*_ values. We plot these mean quantities, one from solving the ODE and other from the mass functionals against one another as in Figure 4. The green line depicts *y* = *x* line in all the six subplots. For a perfect match between the ODE and mass results quantities, the points would lie along this line. We observe a good match between the results for both kinds of masses for ⟨FR⟩ and ⟨*V*_*m*_⟩ with error of less than 10% for most of the firing range of the ODE models. There are a few exceptions, however, at the onset of firing and at the DB onset, where the output of the ODE model is not exactly approximated by the mass functionals.

### 3.2 Dynamics of the Coupled Model: Ordinary Differential Equation Formulation

Using the methodology as described in Coupled Model section in Methods, we couple the two kinds of masses to each other, so as to study the dynamical behaviour of the E − I couplet. Here we investigate the system behaviour in terms of the E and I mass firing rates variations in potassium ion concentration, injected current and coupling strength between the masses. Hence, the mean FR of each of the masses is a function of total current expressed as combination of injected current and the mean FR of the other masses that it couples to. The ODEs of the E − I coupled model are expressed in terms of *FR*_*E*_ and *FR*_*I*_ as:

$$\dot{FR_{E}}=\left(\Phi_{E}\left(I_{E}\left(t\right)\right)-FR_{E}\right)/\lambda_{E}$$ (29)

$$\dot{FR_{I}}=\left(\Phi_{I}\left(I_{I}\left(t\right)\right)-FR_{I}\right)/\lambda_{I}$$ (30)

This form, which is a discretized version of the Wilson-Cowan model (1972) (Wilson and Cowan, 1972), was adopted to acknowledge that firing rates - which are an observation of the system - cannot change instantaneously. *λ*_*E*_ and *λ*_*I*_ are the time constants of the respective masses. The functions Φ are FR functions of the total currents to these masses, as given in Eqs. 16a and 19a. *I*_*E*_(*t*) and *I*_*I*_(*t*) are the total contributions to the E and I masses, respectively, expressed as:

$$I_{E}\left(t\right)=I_{E,inj}+I_{E,pump}-g_{I,E}h_{I}\left(t\right)$$ (31)

$$I_{I}\left(t\right)=I_{I,inj}+I_{I,pump}+g_{E,I}h_{E}\left(t\right)$$ (32)

where the currents *I*_*x*,*inj*_ denote the input currents from outside the network, *I*_*x*,*pump*_ denotes the pump currents, and the coupling constants *g*_*xy*_ and synaptic functions *h*_*x*_(*t*), and the differential equations that govern them, follow the definitions in Section 2.4. Note that the sign of these synaptic inputs are written out explicitly, with inhibitory input appearing as a negative current.

The values of the parameters used for the simulations are, in ms, *λ*_*E*_ = *λ*_*I*_ = 1, *τ*_*E*_ = 10, *τ*_*I*_ = 20, which corresponds to slow (GabaB-type) synapses. Nominal coupling constants used here are ${\hat{g}}_{EI}=0.2$ and ${\hat{g}}_{IE}=0.04$ . Excitatory to inhibitory coupling was kept at the nominal value, and inhibitory to excitatory coupling was varied with respect to the nominal value. Additionally, no external input was applied to the inhibitory mass *I*_*I*,*inj*_ = 0.

**FIGURE 5:**
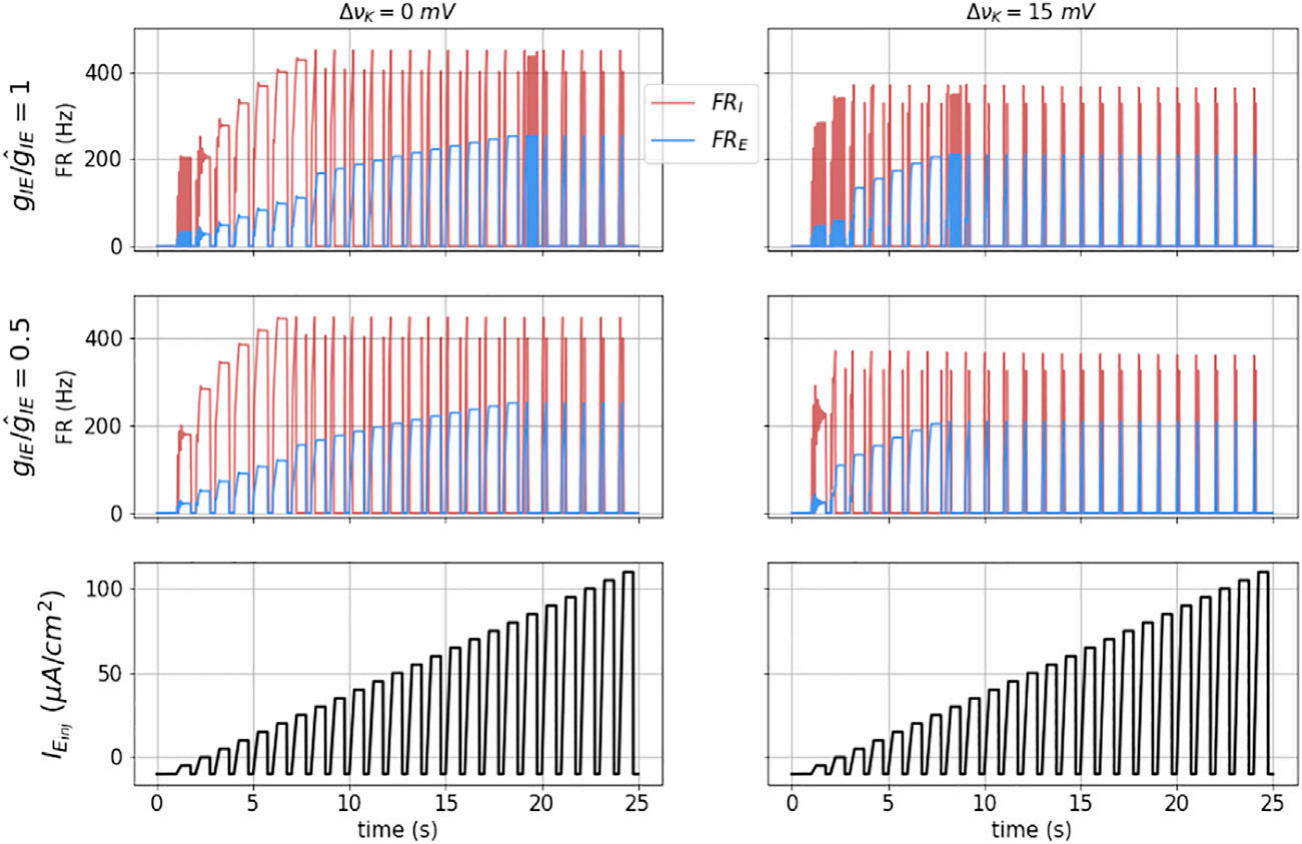
Dynamics of the coupled model: The figures show the dynamical response of the coupled masses in terms of their mean firing rates (FR_*E*_ and FR_*I*_) for a square wave stimulus (shown in black in the bottom most subplots) for Δ*ν*_*k*_ = 0 (left) and Δ*ν*_*k*_ = 15 (right). The top subplots are for nominal I to E coupling strength $g_{IE}={\hat{g}}_{IE}$ and the middle subplots show the dynamics when $g_{IE}=\frac{{\hat{g}}_{IE}}{2}$ .

Some of the fundamental dynamics of this E − I network, under external injected current input *I*_*E*,*inj*_, are shown under four different state conditions in Figure 5. Here the external input is a series of current pulses with a 250 ms ramp input, and 0.75 duty cycle, with increasing amplitudes (lower traces). The upper left panel corresponds to nominal *ν*_*K*_ and *g*_*IE*_. At very low pulse height, this small network oscillates. We expect that addition of fast (GABA-A) inhibition could be used to tune that behavior.

At moderate pulse height, we observe balanced firing of both the excitatory and inhibitory masses. As the pulse height increases, the inhibitory mass is driven into DB (at approximately time *t* = 12 s). At this point the excitatory mass fires at an even higher rate, and is equivalent to the Run-away excitation (RAE). At even higher pulse height, the excitatory mass is driven into depolarization block and both their firing rates go to zero during the pulse. We denote this network state as DB. Note that upon pulse initiation, because the pulses have a sloped front end, both masses initially fire before reaching DB.

In the left, middle panel, we have decreased *g*_*IE*_ by a factor of two. The result is that the transition to RAE occurs at a lower pulse input height.

In the right column we show the results for slightly elevated extracellular potassium (Δ*ν*_*K*_ = 15 mV, or ∼75% increase). In this case the transitions to both RAE and DB occur at lower input currents, are further compressed with decreased *g*_*IE*_.

**FIGURE 6:**
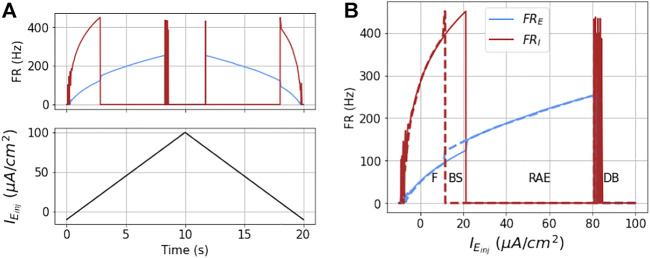
Dynamics of the coupled model to ramp stimulus: The figures show the dynamical response of the coupled masses in terms of their mean firing rates (FR_*E*_ and FR_*I*_) for a ramp stimulus (shown in black in part **(A)**) for Δ*ν*_*k*_ = 0 and $g_{EI}={\hat{g}}_{EI}/2$ . As the injected current to the E mass increases the firing rates increases proportionally, until the I mass goes into DB, which results in unbalanced firing of E mass or run-away excitation. As the current is further increased the E mass goes into DB, at around $I_{E_{\mathrm{inj}}}=81\;\mu A/cm^{2}$ . This is also accompanied by a simultaneous spike in FR_*I*_, owing to momentary drop in current (from its DB threshold) it received from E mass firing. This non-zero FR_*I*_ is not sustained any further as the E mass goes into DB due to very high injected current. This is repeated in reverse as the current goes down from its maximum value in the second half of the stimulus. However, the point at which the I mass recovers from DB (during second half of the current), and at which it goes into DB (in the first half) are not exactly same. These hysteretic dynamics result in bi-stable (BS) region as is shown with the other three regions (F, RAE, and DB) in part **(B)** as a function of injected current.

The network dynamics are hysteretic with respect to injected current. This is apparent from the detailed response of the model to a symmetric ramp current function as shown in Figure 6. Here the output is not the same for increasing input as for decreasing input. A first indication of this is that the ramp points where the I mass goes in and out of DB occur at different values of $I_{E_{\mathrm{inj}}}$ . This is better observed in Figure 6B where the firing rates are plotted as a function of $I_{E_{\mathrm{inj}}}$ , and the sign of ${\dot{I}}_{E_{\mathrm{inj}}}$ is denoted with solid (positive) or dashed (negative) line types. Therefore, in state space, there is bistable region in which the network can either be in stable firing (F) or RAE. We denote this region as BS.

### 3.3 Analytical Boundaries of the IE Network Dynamics

As explored in the previous section, the coupling of the E mass and I mass models can result in four dynamical states as defined not-firing NF, stable firing F, RAE, and DB, and potentially can have bistable state-space regions that can have either. In this section, we delineate analytically the boundaries between these behaviors in parameter-space composed of Δ*ν*_*K*_, *g*_*IE*_, and *I*_*E*,*inj*_.

In the NF state, the masses do not fire due to insufficient total current for E mass firing. In the DB state the E mass does not fire because of very high total current. In the RAE state the E mass shows uncontrolled (pathological) firing due to failure of the I mass to inhibit its firing. This in turn is because the E mass’s firing rate is high enough to put the I mass into DB. In the BS region, the E mass shows two stable firing patterns for the same injected current depending on whether the I mass is in DB or not - and this depends on history. When the I mass is in the F state and injected current is on, the E mass shows one FR (lower), while when I mass is in the DB state, and the same injected current is still on, the E mass shows another FR (higher). This property marks the bi-stable region in the $I_{E_{\mathrm{inj}}}$ - Δ*ν*_*K*_ bifurcation space. Also, at the boundaries of the bi-stable region, the FRs of the E mass are the same. The F state corresponds to the E mass normal firing.

**FIGURE 7:**
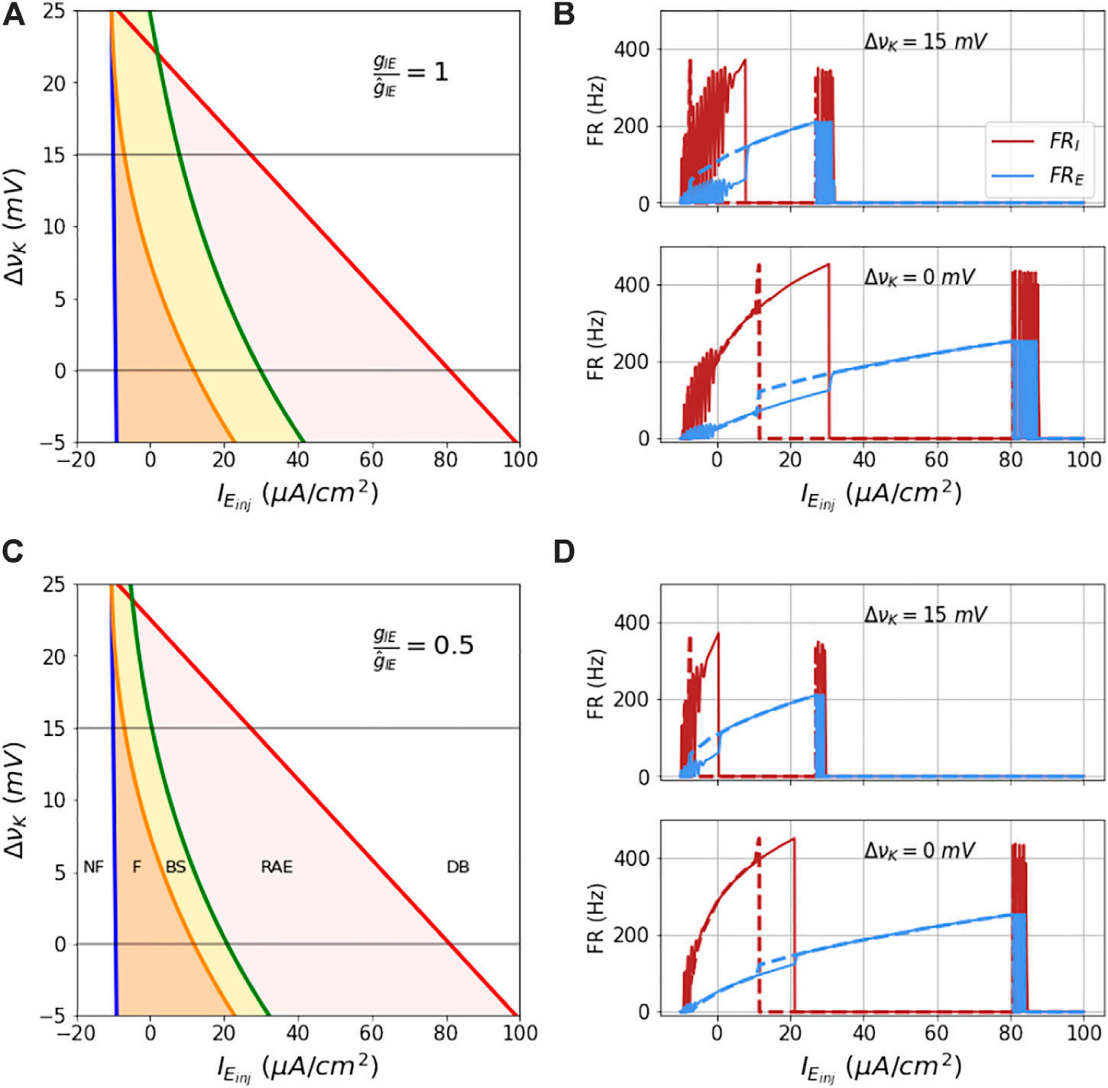
Analytical bounds of dynamical regimes of the coupled model: The figures **(A–D)** show the analytical boundaries for not firing, firing, bi-stability, run-away excitation and depolarization block regions as a function of couplings *g*_*IE*_ and Δ*ν*_*K*_. The boundaries and regions enclosed by them for a range of values of Δ*ν*_*K*_ at two fixed couplings *g*_*IE*_ are shown in **(A,C)**. **(B,C)** show the firing rates of the two masses as a function of ramp current at the two Δ*ν*_*K*_ values 0 and 15 mV (marked in grey line in parts **(A,C)**). The solid lines are firing rates for the increasing current and the dotted lines show the firing rate for the decreasing current.

The analytical boundaries of these regions are shown in Figure 7. Note that in absence of coupling to the I mass, the blue and red lines in the figure enclose the unstable fixed points region of the E mass. On the left of the blue boundary, the state is NF, the state beyond the red boundary is DB. When the coupling between E and I mass is on, the orange region limited by the blue line (firing boundary) on the left and orange line (bistability boundary) on the right is the firing region (F state). The yellow region between the orange and the green line (RAE boundary) is the bistable region (BS state). The red region between the green line and the red line (DB boundary) is the RAE region.

Steps to obtain the Firing onset (FO), Bi-stability (BS), Run-away excitation (RAE) and Depolarization block (DB) boundaries are calculated as follows, where the Nernst potentials are adjusted using Eq. (13).

1) The FO boundary is obtained using the parameters to the functional fits as in the equation of Eq. 15a:

$$i_{E_{\mathrm{th1}}}=a_{E_{\mathrm{FRT}}}\nu_{K_{E}}+b_{E_{\mathrm{FRT}}}$$

2) The RAE boundary is characterized by a point where *I* mass goes into depolarization, after reaching its maximum firing rate and as a result the E mass fires in uninhibited manner. Hence, we first find the total current to the I mass (Q_*I*_) that results in its maximum FR using Eq. 16a.

$$Q_{I}={\left(\frac{FR_{I}}{M_{1}^{I}}\right)}^{2}$$ (33)

Using the obtained Q_*I*_, and Eq. 18, we find FR_*E*_ for the above Q_*I*_ as below. Notice that there is no injected current to the I mass (0 in the equation below).

$$FR_{E}=\left(1/g_{EI}\right)\left(Q_{I}-0+I_{I_{\mathrm{pump}}}\right)$$ (34)

Using the above obtained *FR*_*E*_, we obtain the total current to the E mass, i.e., *Q*_*E*_ using Eq. 19a,

$$Q_{E}={\left(\frac{FR_{E}}{M_{1}^{E}}\right)}^{2}$$ (35)

Now since we know the total current, we can again use equation. 18, to find injected current which is our *I*_*RAE*_, as follows.

$$I_{\mathrm{RAE}}=Q_{E}+I_{E_{\mathrm{pump}}}+g_{IE}M_{1}^{I}$$ (36)

3) The bi-stability boundary is obtained by invoking the condition: FR_*E*_(BS) = FR_*E*_(RAE), which implies that *Q*_BS_ = *Q*_RAE_. Using Eq. 18, we arrive at:

$$I_{BS}-g_{IE}FR_{I_{BS}}-I_{E_{\mathrm{pump}}}=I_{\mathrm{RAE}}-g_{IE}M_{1}^{I}-I_{E_{\mathrm{pump}}}$$ (37)

Since FR_*I*_ at BS zero, the second term on the left hand side of the above equation goes to zero.

Hence, we arrive at:

$$I_{BS}=-g_{IE}M_{1}^{I}+I_{\mathrm{RAE}}$$ (38)

4) The DB boundary is using the parameters of the fit to the DB threshold as in Eq. 15b.

$$i_{E_{\mathrm{th2}}}=a_{E_{\mathrm{DBT}}}\nu_{K_{E}}+b_{E_{\mathrm{DBT}}}$$ (39)

These four boundaries separate the Δ*ν*_*K*_ vs. $I_{E_{\mathrm{inj}}}$ state space into five regions as shown in Figure 7.

In Figure 7A is shown this state space for the default inhibitory-excitatory coupling value $g_{IE}/{\hat{g}}_{IE}=1$ . As *ν*_*K*_ increases all these boundaries shift to lower $I_{E_{\mathrm{inj}}}$ values. This contraction of the firing and bistable regions upon increase in extracelluar potassium is readily illustrated by the hysteretic dynamics in response to a symmetric ramp function, computed from integrating the ODEs as before, shown in Figure 7B. Note also that the transition times observed in the hysteresis correspond well with the analytically computed boundaries, which in turn assume steady state inputs and dynamics. Note also that at this modestly increased potassium level - equivalent to increasing from [*K*]_*o*_ = 3.5 mM to [*K*]_*o*_ = 6.2 mM - that the region of bistability is very small.

In Figure 7C is shown this state space for the decreased inhibitory-excitatory coupling value $g_{IE}/{\hat{g}}_{IE}=0.5$ , with accompanying hysteresis curves for ramp input shown in Figure 7D. Although at nominal extracellular potassium the input current needed to drive the network into DB is only modestly lower (about 18% lower) than for the nominal coupling, at the elevated potassium level is lowered by another factor of about 30% compared to the nominal coupling at elevated potassium.

**FIGURE 8:**
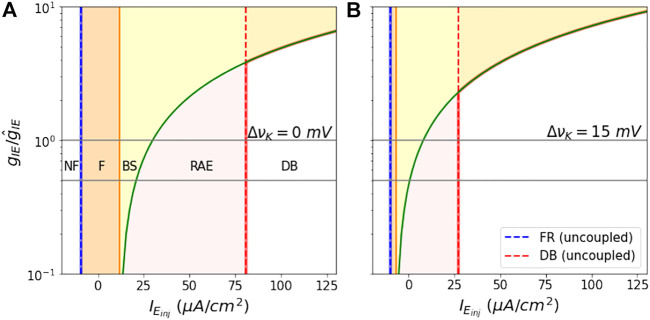
Shift in analytical Bounds as a function of coupling constant: The figures show the shift in dynamical region boundaries as the ratio $\frac{g_{IE}}{{\hat{g}}_{IE}}$ is varied from 0.1 to 10, for Δ*ν*_*k*_ = 0 *mV* in **(A)**, and Δ*ν*_*k*_ = 15 *mV* in **(B)**. The grey horizontal lines depict the two coupling constants for which the region boundaries are shown in Figures 7A,C. The dotted blue and red lines in both the figures represent firing onset and DB onset boundaries of the E mass, when it is not coupled to the I mass.

The state space as a function of $g_{IE}/{\hat{g}}_{IE}$ vs. $I_{E_{\mathrm{inj}}}$ is shown in Figure 8 for values of Δ*ν*_*K*_ = 0, 15 mV. Here again the boundaries between behaviors are smooth, monotonically increasing functions. Higher *ν*_*K*_ leads to smaller areas of the dynamical regions F, BS, and RAE. Beyond the point where the RAE boundary (in green) crosses the DB boundary, the E mass goes into DB right after bistablity. Hence, we shift the DB (in red) boundary to the RAE boundary in the figures.

We note that because of the smooth monotonically increasing shape of these boundaries, that the choice of our nominal value for ${\hat{g}}_{IE}$ and comparison with a halved value would have lead to the same pattern of decreases in region space as discussed in the previous paragraphs.

## 4 Discussion and Conclusion

In the present work our aim was to establish and demonstrate a method to develop computational models of neuronal ensembles - neural masses - that stem from biophysical and coupling characteristics of membrane dynamics resolved cellular models of neurons, whose states and output could quantitatively be mapped across modeling scales, whose bifurcations realistically included depolarization block, and whose dynamics realistically bidirectionally couple to the neurons’ environment.

The developed neural mass models are parametrized through simple mathematical functions, show physiologically interpretable behaviors and dynamical transitions from one state to another, as a function of key parameters of neural environment.

In this work, we have demonstrated this development process for both an inhibitory neural mass, based on the Wang-Buzsáki (Wang and Buzsáki, 1996) (WB), and an accommodating excitatory neuron (SEAN), based on a simplification of the Pinsky-Rinzel (Pinsky and Rinzel, 1994) model. The masses are characterized in terms of time-averaged outputs of the corresponding neuron models, and represent the mean dynamics of populations of these neurons.

These mass descriptions can serve as plug-in replacements for existing NM elements used in other models, in that they produce firing rates as a function of synaptic input from other masses.

These masses also receive as input slow variables in the form of extracellular potassium, for both, and accommodation state for SEAN, and as output provide the coupling to those slow variables in the form of average membrane potential and transmembrane in our models, instead of using a sigmoidal function as the base relation between the input and firing rate, we use a fit for actual firing rates measured from ODE implementations of the original compartment models with each’s firing range, and explicitly the thresholds for firing and depolarization block as a function of the slow variables.

Inclusion of depolarization block has not been included in the classic NMMs (Freeman, 1972a; Freeman, 1972b; Wilson and Cowan, 1972; Lopes da Silva et al., 1974; Freeman, 1975; Jansen and Rit, 1995; Cona et al., 2014) before. Depolarization block was introduced into continuum Wilson-Cowen dynamics (Meijer et al., 2015) in a roughly ad-hoc fashion, but not dependent on [*K*]_*o*_.

The explicit parametrization of depolarization block enables these new implementation of neural masses to express the underlying mechanisms of depolarization blocks and runaway excitation (McCormick and Contreras, 2001) needed for networks built from these elements to express realistic transitions to seizure and spreading depolarization. The sensitivity of both firing rates responses and DB transitions to extracellular potassium concentration then embodies a critical components that are known to play roles in epileptic dynamics and demonstrates the method to incorporate related volume transmitted signaling (Agnati et al., 1992).

Because these mass elements are built to parameterize these from the original cellular models, these variables map directly to measurable variables in brain. We investigated the dynamics of a simple two element network formed from coupling an excitatory and an inhibitory mass in which input was delivered only to the excitatory mass. This network itself displays key features that underlie epilepsy. In particular, as a function of input to the network it transitions in a hysteretic manner from firing to runaway excitation to depolarization block. Importantly, as extracellular potassium is increased, the input threshold to unstable dynamics is decreased. More importantly, as the inhibitory coupling to the excitatory network is decreased, this sensitivity to increases in extracellular potassium is greatly enhanced. This latter observation is consistent with the observation that epileptic networks are not seizing all the time, but are more susceptible to seizures and spreading depolarizations.

### 4.1 Limitations of the Model

The dynamics of the masses we developed are computationally efficient and can be used as direct plug-ins in existing NMM networks, yet their properties (firing rate vs input and environment) are closer to realistic.

This manuscript was built from two well known and established Hodgkin-Huxley style compartment models—that by Wang-Buzsáki (Wang and Buzsáki, 1996) and a reduced version of the Pinsky-Rinzel model (Pinsky and Rinzel, 1994) of a pyramidal neuron. This was done not because these models are the most accurate, but because they are recognizable and understood.

With this choice comes the limitations of these models. For example, we achieve firing rates from our SEAN model that far exceed the maximum firing rate of read pyramical neurons, which is a feature also noted in (Pinsky and Rinzel, 1994) for their soma-only reduction. In addition, by reducing this to a single compartment model we have eliminated burst-firing from this model as it is more complex.

We note that both the WB and SEAN models follow canonical Type 1 transition behavior, with roughly square-root firing rate behavior following. In our modeling, we have only parametrized that behavior and the subsequent transition to depolarization block. We leave for future work parametrization of the host of different transitions that have been articulated for example in (Izhikevich, 2000). Such efforts will further need to deal with the relationship between within burst dynamics and rate of axonally transmitted action potentials and resulting post-synaptic current generation. Likewise, a method to deal with hysteretic firing onset transitions such as observed with the simplest type 1 neurons (Izhikevich, 2000) may be needed.

For the current generated NMMs, we have parametrized the average response of a population of homogeneous neuron types formed with identical detailed parameters. Even when subdivided into common cell types, neurons in real biology will have a distribution of shapes, sizes and even ion channel or pump densities. These different within cell differences will lead to changes in the detailed input/output dynamics that we have parametrized in our models. Given a distribution of such parameters, it is straightforward to build the average mass response based on an average of the parameterized responses given such cell-parameter variations. As long as such cell-parameter variations do not change the firing rate from the square-root shape observed, these will translate simply to shifts in the positions of *ISS*_*F*_ and *ISS*_*DB*_ and the maximum firing rate as function of *ν*_*K*_. As illustrated in Supplementary Figure S4, such averages primarily smooth out the distinct transitions at firing onset and DB offset. We do not expect that such changes will substantially alter the hysteretic network dynamics of firing, runaway excitation, and depolarization block. We anticipate the hardest part of incorporating the heterogenetity into such models is to establish and justify what distribution of cell parameters are realistic and should be used.

### 4.2 Future Directions

An interesting future work with these neural masses will include linking them to spatial networks of extracellular space that include tracking and diffusion of potassium as well as glial buffering and cell swelling. These elements should reveal components that spread seizure and SD events as well as express normal physiological rhythms. Through the procedure used to include other slow variables including intracellular sodium concentration, synaptic vesicle reserve, and oxygen dependent ATP production for driving the ionic pumps, it will also be possible to investigate the role of mutated channel dynamics at the network level.

## Data Availability

All data was generated within the publicly available code contained within the article.
