# Supplementary material for: Development of Mechanistic Neural Mass (mNM) Models that Link Physiology to Mean-Field Dynamics
Source: Front Netw Physiol. 2022 Sep 28;2:911090. doi: 10.3389/fnetp.2022.911090 (PMC9980379; doi:10.3389/fnetp.2022.911090)
Supplement: Supplementary file 1 [file DataSheet1.pdf]

Development of mechanistic neural mass (mNM) models that link physiology to mean-field dynamics

Richa Tripathi[1,2,3], **Bruce J. Gluckman**[1,4,5,*]

[1] *Center for Neural Engineering, The Pennsylvania State University, University Park, Pennsylvania 16802, USA*
[2] *Center for Advanced Systems Understanding (CASUS), HZDR, 02826 Görlitz, Germany*
[3] *Indian Institute of Technology Gandhinagar, Gujarat, India*
[4] *Departments of Engineering Science and Mechanics, Biomedical Engineering, The Pennsylvania State University, University Park, Pennsylvania 16802, USA*
[5] *Department of Neurosurgery, College of Medicine, The Pennsylvania State University, Hershey, Pennsylvania 17033, USA*

Correspondence*:
Bruce J. Gluckman
BruceGluckman@psu.edu

This work is supplementary text for the article of the same title by Tripathi and Gluckman (2022).

SUPPORTING INFORMATION

Parameters for the Wang-Buzsáki and SEAN models

For the WB neuron model of inhibitory neurons, the activation functions, model equations and the parameter values are taken from the original paper (Wang and Buzsáki, 1996). For the SEAN model, we used a single compartment version of Pinsky-Rinzel model (Pinsky and Rinzel, 1994), where we also retain the potassium hyper-polarization current (of the dendritic compartment). The parameters of both models are listed in Table 1.

WB	SEAN
$g_{Na} = 35$	$g_{Na} = 30$
$g_K = 9$	$g_K = 15$
$g_L = 0.1$	$g_L = 0.1$
$\psi = 5$	$g_{K-AHP} = 0.8$
$C_m = 1$	$C_m = 3$
$\nu_L = -65$	$\nu_L = -60$
$\nu_{Na} = 55$	$\nu_K = 60$
$Na_i = 25$	$Na_i = 25$
$V_{1_W} = 35$	$V_{1_S} = 46.9$
$V_{2_W} = 60$	$V_{2_S} = 19.9$
$a_{1_W} = 0.1$	$a_{1_S} = 0.32$
$b_{1_W} = 10.0$	$b_{1_S} = 4$
$a_{2_W} = 4$	$a_{2_S} = 0.28$
$b_{2_W} = 18$	$b_{2_S} = 5$
$V_{3_W} = 34$	$V_{3_S} = 24.9$
$V_{4_W} = 44$	$V_{4_S} = 40$
$a_{3_W} = 0.01$	$a_{3_S} = 0.016$
$b_{3_W} = 10.0$	$b_{3_S} = 5$
$a_{4_W} = 0.125$	$a_{4_S} = 0.25$
$b_{4_W} = 80$	$b_{4_S} = 40$
$V_{5_W} = 58$	$V_{5_S} = 43$
$V_{6_W} = 28$	$V_{6_S} = 20$
$a_{5_W} = 0.07$	$a_{5_S} = 0.128$
$b_{5_W} = 20$	$b_{5_S} = 18$
$a_{6_W} = 0$	$a_{6_S} = 4$
$b_{6_W} = 10$	$b_{6_S} = 5$

Table 1. Parameter sets of the WB and SEAN ODE models.

Mechanistic Neural Mass parametrization parameters

The mass model parameters governing the form of the functions for $\langle \mathrm{FR} \rangle$, $\langle K_{Flux} \rangle$, and $\langle \mathrm{V}_m \rangle$, for both the inhibitory and the excitatory masses are listed in the Table 2.

I mass	E mass
$a_{I_{FRT}} = 0.014$	$a_{E_{FRT}} = -0.045$
$b_{I_{FRT}} = 1.746$	$b_{E_{FRT}} = -12.705$
$a_{I_{DBT}} = -0.835$	$a_{E_{DBT}} = -3.591$
$b_{I_{DBT}} = -50.621$	$b_{E_{DBT}} = -188.312$
$a_{I_{FR}} = -0.005$	$a_{E_{FR}} = -0.039$
$b_{I_{FR}} = -1.5$	$b_{E_{FR}} = -8.121$
$c_{I_{FR}} = -141.837$	$c_{E_{FR}} = -135.304$
$d_{I_{FR}} = -4110.487$	$a_{E_{V_m}} = -0.001$
$a_{I_{V_m}} = -0.007$	$b_{E_{V_m}} = -0.265$
$b_{I_{V_m}} = -1.368$	$c_{E_{V_m}} = -46.526$
$c_{I_{V_m}} = -95.054$	$a_{E_{KF}} = -0.002$
$a_{I_{KF}} = -0.903$	$b_{E_{KF}} = -1.536$
$b_{I_{KF}} = -32.702$	$c_{E_{KF}} = -53.984$

Table 2. Parameter sets of the E and I mass thresholds determination

The means of dynamical quantities (FR, K_{flux}, and V_m) from the simulations of the neuron models for a range of injected currents and different values of potassium Nernst potentials are shown in Supplementary Figure S1.

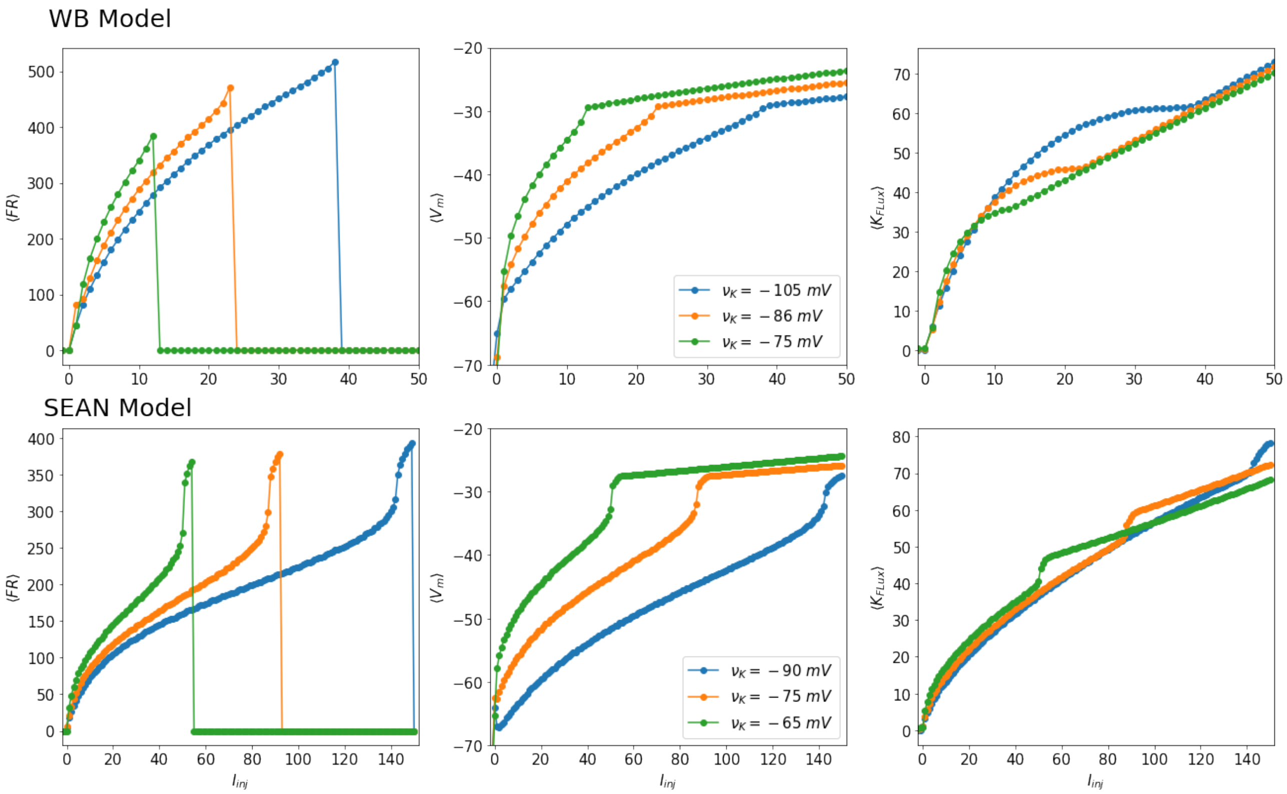

Supplementary Figure S 1. Neuron Model Outputs: The figures show the $\langle \text{FR} \rangle$, $\langle V_m \rangle$, and $\langle K_{FLux} \rangle$, of the WB and SEAN models, as a function of injected currents at three different values of ν_K depicted in the middle subplots. Notice the discontinuous and very high $\langle \text{FR} \rangle$ of the SEAN model just before the DB region. Because of this discontinuity, we parameterize the DB threshold of the E neural mass at $\text{ISS}_{DB} - \Delta$.

The firing threshold, DB threshold, maximum $\langle \text{FR} \rangle$s, maximum $\langle \text{V}_m \rangle$, and maximum $\langle \text{K}_{flux} \rangle$, and the best fits to them, for both the neuron models as a function of ν_K are shown in Supplementary Figure S2, and Supplementary Figure S3, respectively.

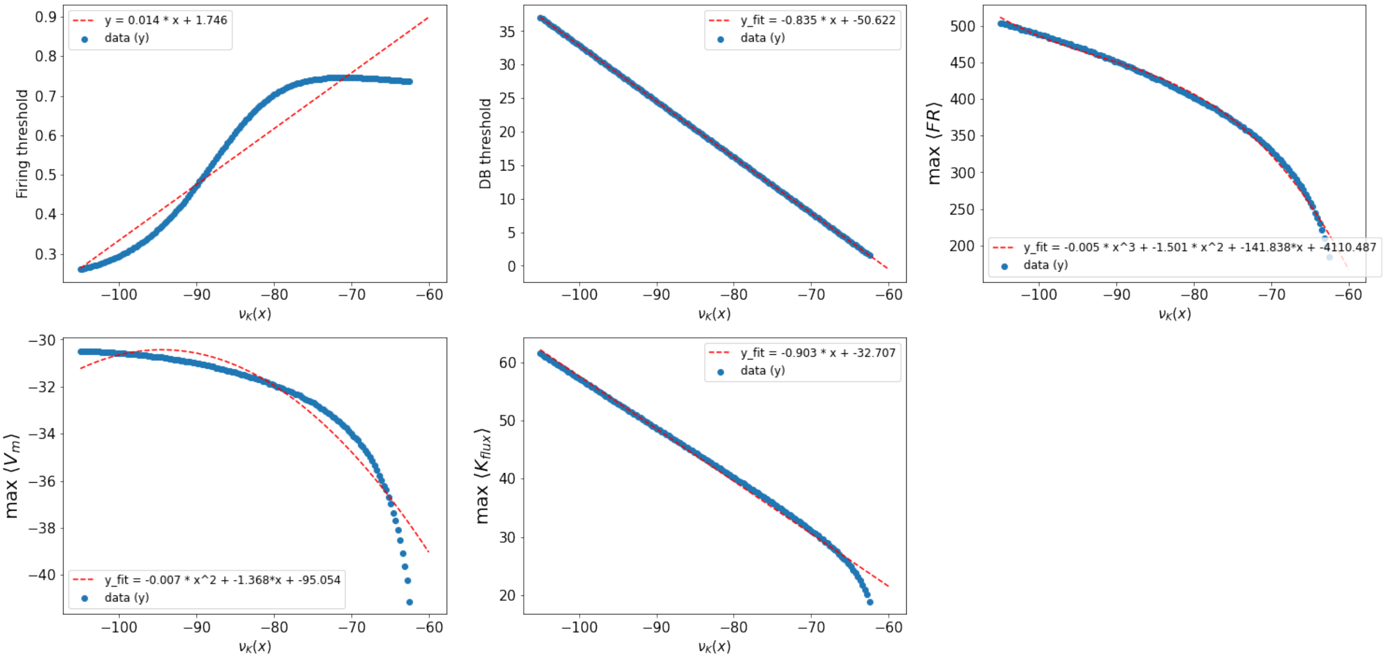

Supplementary Figure S 2. Analytical fits to WB model thresholds: The figures show best analytical fits (in red) to firing threshold, DB threshold, maximums of mean FR, mean K_{Flux}, and mean V$_m$ of the WB neuron model shown in blue.

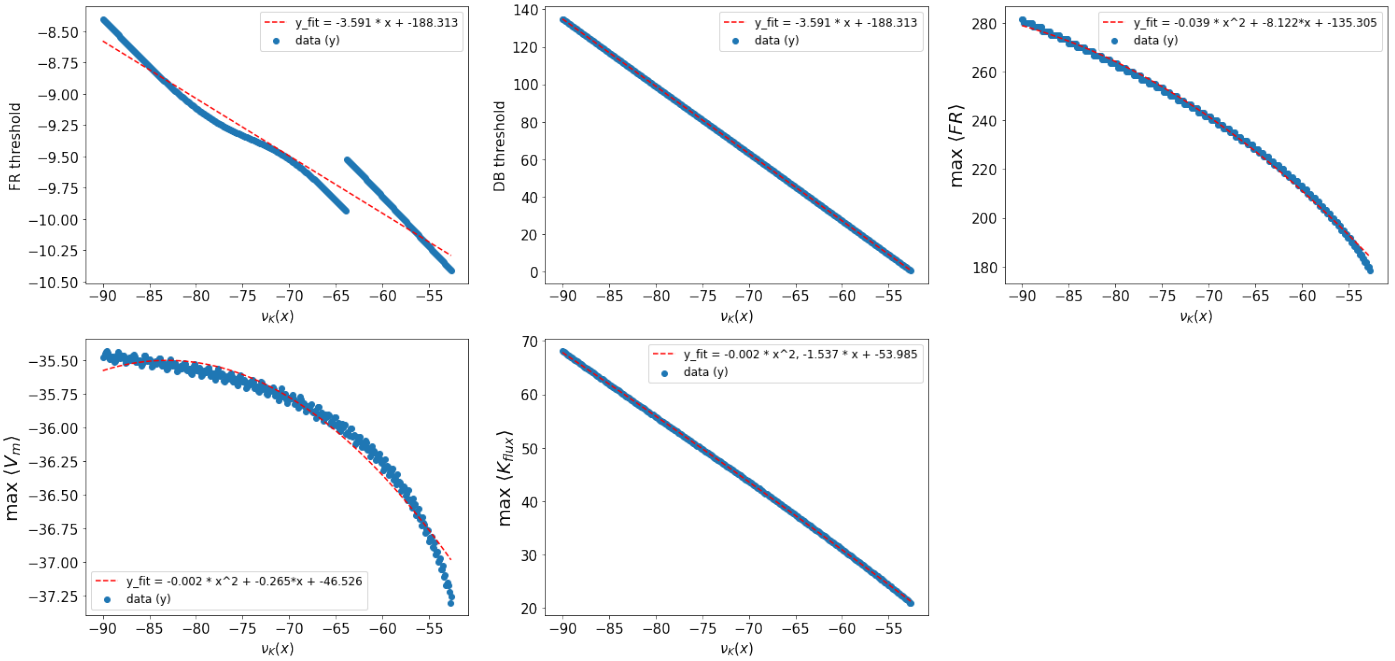

Supplementary Figure S 3. Analytical fits to SEAN model thresholds: The figures show best analytical fits (in red) to firing threshold, DB threshold, maximum values of mean FR, mean K_{Flux}, and mean V$_m$ of the SEAN neuron model shown in blue.

Method for incorporating parameter heterogeneity in the neuronal populations

The neural masses, in our manuscript, represent homogeneous populations of neurons. We show here, how can we incorporate heterogeneity in a straight-forward manner and how does it affect the functions for mean dynamical quantities. For the nominal Nernst potential values, we obtain distributions of firing

onset, DB and maximum FR values from uniform distributions. The *mean* of the distribution were the actual values of the quantities as obtained from our functions in the main text, and the extent of distribution ($d = 5$) was chosen to be in range ($[mean - d, mean + d]$). Then we obtained the $\langle \text{FR} \rangle$ values for a range of currents from equations in the main text, for each combination of the values from distribution. Finally we averaged over all the curves (1000 combinations) and plotted it as a function of current as shown in Supplementary Figure S4. We observed that for most of the dynamic range, and for both the masses, the average $\langle \text{FR} \rangle$ is the square root function of current, except at the bounds where it seems to be sigmoidal.

This approach has been used elsewhere to produce NMMs for heterogeneous masses (see for example Stefanescu and Jirsa (2008); Rodrigues et al. (2010); Deschle et al. (2021)), but these efforts start from simplified single-neuron elements such as FitzHugh-Nagumo and Hindmarsh-Rose and integrate and fire models whose dynamics do not have the mechanistic components we sought to embody here. The systematic incorporation and study of impact of of population heterogeneity in our model is a part of the future work.

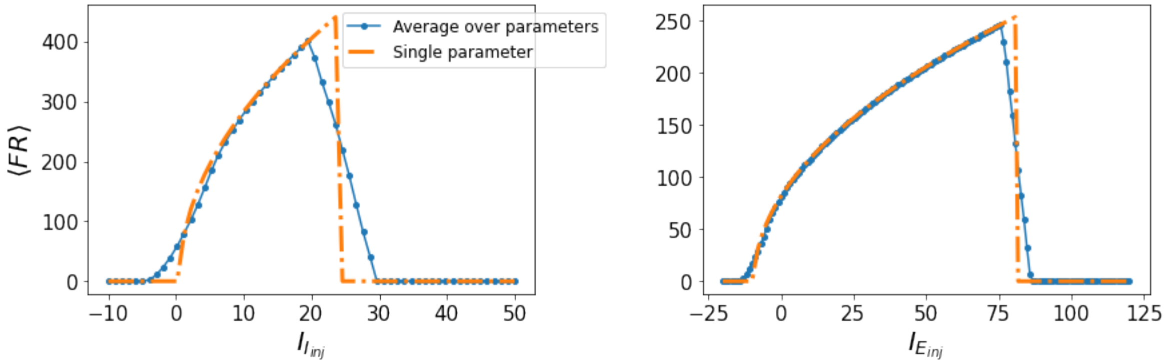

(a) (b)

Supplementary Figure S 4. Neuron population heterogeneity: The figures show a comparison of the $\langle \text{FR} \rangle$s for a single neuron (WB (a) and SEAN (b)) characterized by a single parameter combination (firing onset, DB onset and maximum FR), and an average over 1000 parameter combinations chosen from a uniform distribution. On the x-axis are the injected currents for the WB model neurons (a), and for SEAN neurons (b).

REFERENCES

Deschle, N., Ignacio Gossn, J., Tewarie, P., Schelter, B., and Daffertshofer, A. (2021). On the validity of neural mass models. Frontiers in computational neuroscience , 118

Pinsky, P. F. and Rinzel, J. (1994). Intrinsic and network rhythmogenesis in a reduced Traub model for CA3 neurons. Journal of computational neuroscience 1, 39–60

Rodrigues, S., Chizhov, A. V., Marten, F., and Terry, J. R. (2010). Mappings between a macroscopic neural-mass model and a reduced conductance-based model. Biological cybernetics 102, 361–371

Stefanescu, R. A. and Jirsa, V. K. (2008). A low dimensional description of globally coupled heterogeneous neural networks of excitatory and inhibitory neurons. PLoS computational biology 4, e1000219

Tripathi, R. and Gluckman, B. J. (2022). Development of mechanistic neural mass (mNM) models that link physiology to mean-field dynamics. Frontiers in Network Physiology doi:10.3389/fnetp.2022.911090

Wang, X.-J. and Buzsáki, G. (1996). Gamma oscillation by synaptic inhibition in a hippocampal interneuronal network model. Journal of neuroscience 16, 6402–6413
